# Unlocking the potential of smartphone and ambient sensors for ADL detection

**DOI:** 10.1038/s41598-024-56123-0

**Published:** 2024-03-05

**Authors:** Marija Stojchevska, Mathias De Brouwer, Martijn Courteaux, Bram Steenwinckel, Sofie Van Hoecke, Femke Ongenae

**Affiliations:** IDLab, Ghent University-imec, Zwijnaarde, Belgium

**Keywords:** Human activity recognition (HAR), Real-world data, Smartphone sensor data, Ambient sensor data, Computer science, Quality of life

## Abstract

The detection of Activities of Daily Living (ADL) holds significant importance in a range of applications, including elderly care and health monitoring. Our research focuses on the relevance of ADL detection in elderly care, highlighting the importance of accurate and unobtrusive monitoring. In this paper, we present a novel approach that that leverages smartphone data as the primary source for detecting ADLs. Additionally, we investigate the possibilities offered by ambient sensors installed in smart home environments to complement the smartphone data and optimize the ADL detection. Our approach uses a Long Short-Term Memory (LSTM) model. One of the key contributions of our work is defining ADL detection as a multilabeling problem, allowing us to detect different activities that occur simultaneously. This is particularly valuable since in real-world scenarios, individuals can perform multiple activities concurrently, such as cooking while watching TV. We also made use of unlabeled data to further enhance the accuracy of our model. Performance is evaluated on a real-world collected dataset, strengthening reliability of our findings. We also made the dataset openly available for further research and analysis. Results show that utilizing smartphone data alone already yields satisfactory results, above 50% true positive rate and balanced accuracy for all activities, providing a convenient and non-intrusive method for ADL detection. However, by incorporating ambient sensors, as an additional data source, one can improve the balanced accuracy of the ADL detection by 7% and 8% of balanced accuracy and true positive rate respectively, on average.

## Introduction

The population is ageing, resulting in a shift from acute to chronic care where people are living longer with one or more chronic diseases, requiring more complex care. Keeping our elderly physically, mentally, and socially active and independent in their own homes, while also timely being able to respond to care needs, poses a challenge to the healthcare system, which is already dealing with increased financial pressure and staff shortages, which need to be carefully addressed. As a result, in recent years, we have seen a trend of prioritising homecare (or ‘aging in place’) over residential care^1^. The concept of ‘Aging in place’ aims to facilitate elderly people to remain in their homes for as long as possible. Many studies have shown that seniors not only prefer to age in place, but that it also benefits their quality of life (QoL)^2–4^. Ambient assisted living (AAL) is a concept that is often used to address this healthcare challenge^2^. A typical AAL system implements user behavior monitoring and provides a mechanism for alerting family or caregivers in a situation of emergency or an alarming deviation in behavior^2,5^.

User behavior monitoring typically involves observing an individual’s activities and identifying any deviations or restrictions in their behavior. To be able to implement a reliable behavior monitoring system, there is a need for data acquisition and accurate algorithms for detecting activities, based on this data. AAL systems make data acquisition possible by integrating a wide variety of sensors: ambient (e.g., temperature, humidity, light, and motion), object (e.g., magnetic switch for detecting opening of doors or cabinets, and power consumption of electric appliances), video cameras, body-worn motion sensors (e.g., accelerometer integrated in a wrist band) and biosensors (e.g., heart rate and blood pressure sensors)^2^.

As for the detection of activities, in recent years there has been a lot of research^6^ conducted on human activity recognition (HAR), activities of daily living (ADLs) detection, and behaviour modelling. A significant portion of the research today heavily relies on the utilization of multiple sensors placed throughout various rooms and objects. These sensors may include Radio Frequency Identification (RFID) tags on objects, power consumption meters, monitoring systems for ventilation and lighting, as well as cameras strategically installed within the household^7–9^. However, it is important to note that not all sensors are suitable for real-world scenario applications as some sensors may generate low-quality signals or suffer from excessive noise, while others may not be well-received by the elderly due to concerns related to privacy and intrusiveness.

Therefore, when installing an AAL platform, several requirements and limitations on the sensors have to be kept in mind, which is often neglected in existing research^2^. A first requirement is that wearable sensors should be small in size and have low power consumption^2,10^, which is necessary to ensure their practicality and user acceptance. Second, technologies should be cheap and easy to use, otherwise the acceptance rate might drop^11^. Third, the data that these sensors produce should not raise privacy concerns^12^.

Lastly, it is crucial that these sensors generate high-quality data with minimal noise, ensuring that the algorithms utilizing this data can reliably process and interpret it. Therefore, there is a pressing need for research on HAR and ADL detection based on sensors that fulfill the above requirements and can cope with the following limitations: they should be lightweight, easy to use, and unobtrusive to get a high user acceptance.

At the same time, smartphones are equipped with multiple sensors and fulfill these requirements, which makes them adequate devices for real-world and long-term behavior monitoring. A smartphone includes motion sensors, such as accelerometer and gyroscope, a light sensor, and a proximity sensor, among potential others. Smartphone sensor data is nowadays widely used, even among the elderly, and their usage is still growing^13^. For example, with the goal of measuring your vital signs, FibriCheck uses phones for heart rhythm monitoring, or Google Fit monitors your respiratory rate. Phone usage monitoring can also give an indication of the elderly peoples’ cognitive well-being, social inclusion and communication^14^.

Smartphones’ sensors are however not the only sensors that fulfill the discussed requirements. Ambient sensors, such as motion sensors, and magnetic switches, are also appropriate for real-world scenario applications^2^, and have great potential in the area of ADL detection.

Therefore, in this paper, we research the potential of both smartphones and ambient sensors for detecting ADL in real-world scenarios. We take the smartphone as a base device and its sensors’ signals as main source of data and propose a Machine Learning (ML) methodology for ADL detection. We then incorporate other ambient sensors’ data and compare their impact on the accuracy of the obtained models. Through our paper, we tackle the research challenge of developing reliable and accurate ML models for ADL detection. Specifically, our focus is on utilising data from sensors that meet the necessary requirements for real-world scenario applications.

The remainder of this paper is organized as follows: In section “Datasets”, we describe the datasets that are often used in existing research and we discuss their strengths and drawbacks. We then describe the dataset we collected and used in our research, and which we made openly available for future research and analysis. In section “Related work”, we summarize the related work and we discuss the state-of-the-art ML approaches for ADL detection based on smartphone and ambient sensors. In section “Methodology” we describe our proposed research approach, the sensors used and the features explored on this sensor data, as well as the designed ML architecture. In section “Results”, we describe the different experiments performed and according results. In section “Discussion”, we discuss the findings we made with our approach. Finally, in section “Conclusion and future work”, we draw conclusions and discuss future work.

## Datasets

To facilitate the design and evaluation of smartphone-based HAR (SB-HAR) methodologies, many researchers have made an effort to collect and publish datasets. In this section we describe the most relevant datasets, and discuss their strengths and weaknesses. We refer to Table 1, for a summary of the different datasets.

The UCI-HAR^15^ dataset consists of data collected from 30 volunteers. Each of them performed six activities: walking, walking upstairs, walking downstairs, sitting, standing, and lying down. They wore the smartphone on the waist from which accelerometer and gyroscope signals were sampled at 50 Hz. The subjects followed a predefined protocol during data collection.

The WISDM^16^ dataset includes data from 51 subjects. Each of them performed 18 activities for a duration of 3 min each. Aside from coarse activities, such as walking, jogging, and sitting, they also collected ADL, e.g., eating soup, writing, and drinking from a cup. The smartphone was placed in the pocket of the participants.

The UniMiB SHAR^17^ dataset consists of data from 30 subjects performing 9 coarse activities and transitions. The data collection followed a protocol and subjects were asked to place the phone in the front trouser pockets, half of the time in the left and the other half in the right pocket. The activities include, among others, standing up after lying down, running, walking, and going upstairs. The SB-HAR^18^ dataset consists of data from 30 subjects. They performed a protocol of six basic activities (standing, sitting, lying, walking, walking downstairs and walking upstairs). They additionally collected postural transitions: stand-to-sit, sit-to-stand, sit-to-lie, lie-to sit, stand-to-lie and lie-to stand. Participants wore a smartphone on the waist. The experiments were recorded with a video camera, and the recordings were used for labeling the activities and the transitions.

The KU-HAR^19^ dataset consists of data from 90 subjects and 18 different coarse activity classes, such as standing, sitting, walking, taking the stairs, and jumping. The data collection took place in a classroom, which was monitored by the researchers. Participants placed the smartphone in a pouch bag around their waist. The dataset collected by Garcia-Gonzalez^20^ consists of smartphone data from 19 subjects. Unlike the other datasets, which were collected in a controlled environment and following a protocol, this data was collected in the subjects’ own environment and with their own smartphones. The position of the smartphone is also unknown. This dataset however consists of only four activity classes: inactive (meaning not carrying the phone), active (carrying the phone and performing an activity that does not include ambulation, such as sitting), walking (ambulatory activities, such as walking, running and jogging), and driving (moving with engine-powered transport, such as a car, train, bus, or motorbike). The Extra-Sensory^21^ dataset consists of data collected from 60 subjects in their own natural environments, with their own phone and not following any protocol, but rather engaging in their own routine. This dataset includes a large number of activities, both coarse activities, such as walking, standing, and sitting, and ADL, such as cleaning, cooking, and washing dishes. This dataset also includes context data from the phone, such as light, temperature, and audio. A drawback of this dataset is that the data is not continuous, as every minute only 20 s of data are being sampled. Additionally there are mislabeled samples^22^, such as a flat accelerometer signal when the label is walking, as well as impossible combinations of labels, for example walking and sitting at the same time. However, this mislabeling is to be expected as the activities were real-world and free, the study was uncontrolled, and the labels are self-reported by the participants.

The DAHCC^23^ dataset was collected by our research group and consists of data from 31 participants. Data collection was carried out in a house equipped with ambient sensors. Additionally, participants wore a wrist-worn wearable sensor and used a smartphone from which various different data modalities were collected. All the participants were provided with a neck lanyard for their smartphone, which could be attached with a magnet on the t-shirt or blouse of the participant. This was chosen as it is an unobtrusive manner to ensure that the smartphone is carried as much as possible on the person, and many of the elderly are already used to wearing a nurse call button around the neck in this fashion. They were all asked to use the lanyard as much as possible, but were free to not do so if they didn’t want to. Each participant spent a day in the house. During data collection participants were asked to do their own daily routine as if they were at their home. The labels were provided by the participants themselves through a mobile phone labeling application installed on the same phone from which data was collected. The environment was semi-controlled, in the sense that everyone collected data in the same house, but participants were not asked to perform the ADLs in specific places within the house. Execution is however uncontrolled as participants could choose when and where to perform the activities, using objects of their choice, etc. In this dataset there are more than 12 ADL including cooking, showering, working on a computer, eating, toileting and watching TV.

Table 1 highlights the differences between the discussed datasets. Within this research, we evaluated our ADL detection method using the DAHCC dataset because it is the only dataset focusing on ADL, which contains continuous sensor data from both ambient and smartphone sensors. Moreover, the dataset was collected in a real-world environment, in which participants were free to choose their actions as they would in their normal daily life. As such, this give a realistic view on how ADL are performed in the home. Note that one limitation remains of all the aforementioned datasets, including the DAHCC dataset, i.e., they involve adult participants, meaning that no elderly were included, so additional efforts might be needed to translate ADL detection to the elderly population.

**Table 1:** Benchmark datasets used in the literature and their characteristics. Abbreviations: #S (number of subjects), OE (observed execution), VR/VI (video recording / visual inspection), SR (self report) C (controlled), UC (uncontrolled), SC (semi-controlled), P (protocol), F (free).

Dataset	#S	Devices	#Activities	Type of activities	Sensor signal	Labeling procedure	Environment and execution
UCI-HAR	30	Smartphones	8	Low-level	Continuous within activity	OE	C and P
WISDM	51	Smartphones, smartwatches	18	Low-level	Continuous within activity	OE	C and P
UniMiB SHAR	30	Smartphones	9	Low-level	Continuous within activity	OE	C and P
SB-HAR	30	smartphones	6	Low-level	Continuous within activity	VR	C and P
KU-HAR	90	Smartphones	18	Low-level	Continuous within activity	OE	C and P
Garcia-Gonzalez	30	Smartphones	4	Low-level	Continuous	SR	UC and F
ExtraSensory	60	Smartphones, smartwatches	51	ADL	20 s every minute	SR	UC and F
DAHCC	31	Smartphones, smartwatches ambient sensors	12+	ADL	Continuous	SR	SC and F

## Related work

Due to the widespread availability and affordability of smartphones, the use of smartphone sensors for HAR has gained significant attention in recent years, including the exploration of accelerometers, gyroscopes, magnetometers, and/or a combination for these, for HAR. Two types of studies can be discerned. The first focuses on processing the sensor data to classify low-level (coarse) activities, such as walking, running, cycling, and sitting. The second focuses on detecting more complex (ADL) activities, such as cooking, eating and cleaning. In this section, we review the most relevant and recent studies for each of these.

### Low-level activity recognition

There has been extensive research focusing on the detection of low-level activities, such as sitting, standing, walking, running, cycling and taking the stairs.

Shoaib et al.^24^ use a traditional ML approach, in which they use sliding windows over the smartphone sensor data to extract features and then apply a classification algorithm, to identify seven activities: walking, running, sitting, standing, jogging, biking, walking upstairs and walking downstairs. They studied nine different classification algorithms, such as Support Vector Machines (SVM), Random Forest (RF) and Logistic Regression (LR). Performance of the models was compared when using only accelerometer, only gyroscope and the combination of both. They performed all experiments placing the phone on five different locations: right/left jeans pocket, belt position above the right leg, right wrist, and right upper arm. In this work they achieve accuracy of above 90% for all the activities, though they report cross-validation results with stratified split, which means that there might be data from the same subjects in both training and test. They conclude that the accelerometer signal is in general superior to the gyroscope to classify the different activities, except for the stairs activities. Additionally they found that these sensors complement each other only when they both individually perform poorly. If one of the two achieves high accuracy individually, then their fusion brings marginal improvement. Drawbacks of this study are the limited number of activities and the data collection protocol, which is performed in a controlled environment and is not representative for real-world scenarios. The data they collected is not publicly available.

Reyes-Ortiz et al. researched^18^ the detection of not only low-level activities, but also transitions, such as sit-to-stand, lie-to-sit, and lie-to-stand. They also applied a traditional ML approach, calculating features from the accelerometer signal and training a SVM for activity and transition detection. They evaluated their methodology on the SB-HAR dataset which is described in section “Datasets”. Hence, their results may not reflect the performance of their approach in real-world scenarios.

More recently, Skider and Nahid^19^ proposed a RF model for classification. The model is evaluated on the KU-HAR dataset described in section “Datasets”. They achieved an overall accuracy of almost 90%. Similarly to the previous two works discussed, their data was collected in a controlled environment following a protocol, limiting the relevance to real-world applications.

In recent years deep learning techniques have gained more attention due to their capabilities of automatically learning features and higher classification accuracy of human activities in general. For example, Bianchi et al.^25^ propose a convolutional neural network (CNN) architecture. They evaluated the performance of their model on the UCI-HAR dataset, which employed a controlled and observed protocol, and achieved a global accuracy of 92.5%.

Abdel-Basset^26^ proposed a long short-term memory (LSTM) architecture followed by an attention mechanism for the temporal fusion of sensor data. Concurrently they applied a convolutional residual network for the spatial fusion of sensor data. They evaluated their methodology on the UCI-HAR and WISDM datasets, which are both controlled and observed protocols, and achieved results of more than 97% in F-1 score on both datasets.

A CNN-LSTM model is proposed by Ankita^27^, in which the raw signal is used as input for the CNN, and the output of the CNN is used as an input for the LSTM. They evaluated their methodology on the UCI-HAR dataset and achieve a global accuracy of 97.89%.

Even though the vast majority of research is being evaluated on data collected in controlled environment^28^, there are some studies that evaluate their methodology on real-world data.

For example, in the work of Cruciani^22^, the authors proposed a personalization methodology for HAR. They focused on five low-level activities: lying, sitting, walking, running, and cycling. They evaluated their methodology on the Extrasensory^21^ dataset, described in section “Datasets”. Their best performing model achieved a balanced accuracy of 75%, and they observed difficulty in distinguishing sitting from lying activity. For the sitting activity they observed a recall of only 54%.

Collecting labeled real-world data is a cumbersome and difficult task that comes with many challenges. However, it is of crucial importance to train and evaluate models on real-world data, as there is little evidence that models trained on data collected in controlled settings generalizes to real-world settings^28,29^.

In this work, we regard low-level activity recognition as only a utility rather than main focus of the research. Nevertheless, we still encountered challenges to follow the best practices and lessons learned from previous studies. The dataset we used in our research does not contain low-level activity labels. So, instead of using a publicly available data that was collected in controlled setting and train our low-level HAR model on this data, we decided to take an extra step and obtain labels. We achieved this by using the available wrist-worn wearable data and using a pre-trained model, which has previously been evaluated on real-world data, to obtain the labels. This way we could train and evaluate our model on real-world data which was adequate for our research. With this approach we try highlight the importance of using real-world data and to show the community that there can be creative ways to retrospectively obtain labels for unlabeled data.

### ADL recognition

Whereas there is a lot of research on the detection of low-level activities using smartphones, the research on the detection of ADLs is rather limited. There are few studies on ADL detection, such as those of Wanf et al.^30^ and Stikic^31^ that combine wearable (wrist-worn) sensor and ambient sensor data. However, in our work we focused on the potential of a smartphone, and hence keep the scope of related work section also to research using smartphones. To best of our knowledge there is only one work that has focused on using smartphone for ADL detection. The work of Roy^32^ used an indoor location sensor (infrared motion sensor) to provide context for the smartphone-based sensing. To do so, they detected low-level activities from the raw accelerometer data, and associated each ADL with a six-dimensional feature-vector consisting of the number of frames (or duration) of each of the (six) low-level activities. For example, if an instance of cooking consisted of three frames of sitting, four frames of standing and seven frames of walking, the corresponding feature vector would be [3,4,7,0,0,0] as the last three low-level activities (running, lying, and climbing stairs) do not have any occurrences in the instance of cooking. As a last step they added a seventh dimension to this feature vector that represented the indoor location of the person (here limited to kitchen or living room). The ADLs they collected were cleaning, cooking, taking medication, sweeping, washing hands, and watering the plants. Each subject repeated the ADLs four times. In their evaluation they discussed that the low-level activities are not representative for the ADLs they picked, as they did not achieve a classification accuracy higher than 50%. They based their conclusion on the fact that the ADLs they research are very specific and mainly hand involving activities and the low-level activities are either lower body movements or simply body postures. However the feature vector they compute from the low-level activities has also several limitations: they only counted the number of frames in which a low-level activity was detected, but there was no information about the order in which they happened. Additionally they used a hard activity decision for each frame whereas using the probabilities for each low-level activity within that frame might be more informative. They evaluated their approach on a second dataset, namely the Opportunity^33^ dataset. This dataset does not contain smartphone data, but does have data from several body worn accelerometer sensors, complemented with accelerometer and gyroscope sensors placed on objects, and ambient sensors, such as switches and accelerometer sensors in drawers, kitchen appliances and doors. In this dataset there are no explicit low-level activities present, but it does contain micro-movements, such as opening and closing of the fridge and cupboards, toggling the lights on and off, and cleaning the table. They also collected ADLs, such as grooming, preparing and drinking coffee, and preparing and eating a sandwich. Applying the same methodology of inferring ADLs based on micro-movements and location, they observed reasonable results, an average of about 75%, on the Opportunity dataset. They concluded that their approach can be relevant if the low-level sequences are representative for the ADLs. However, they shifted the challenge towards detecting these micro-movements, which might be more challenging than detecting the low-level activities. Additionally the ADLs they considered are rather limited and do not contain crucial activities such as toileting and showering. For the low-level activity classification, they tested several algorithms, i.e. multi-layer perceptron, Naive Bayes, Bayes Net, Decision Table, Best-First Tree and K-star. The paper does not elaborate whether the micro-movements detection was tackled. All of these used a vector of features calculated from the raw accelerometer data. For the location inference they used the Hidden Markov Model (HMM) algorithm.

Currently there is only limited research on ADL detection based on smartphone data. There is a big gap in research regarding different aspects: the smartphone potential hasn’t been explored in great extent, and the methodology is evaluated on lab collected data and is not representative for real-life scenarios. In our work we try to close this research gap by exploring different smartphone modalities. We additionally research the potential of incorporating ambient sensors for more accurate ADL detection. Moreover, the approach we propose is adequate for continuous and long-term ADL monitoring. Finally, we evaluate our models on a real-world collected dataset.

## Methodology

**Figure 1:**
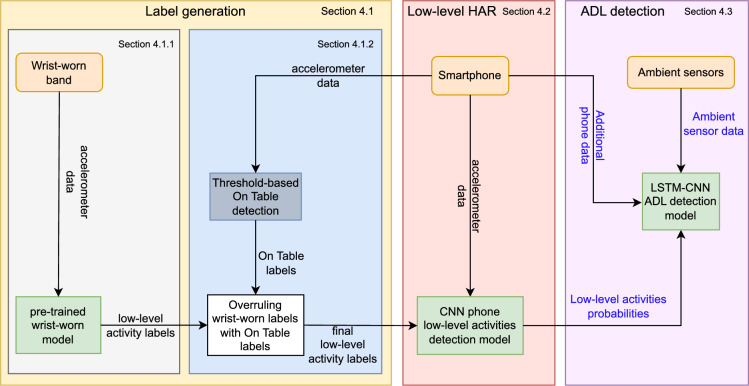
Overview of the different sensor modalities and algorithms that either are used to generate the (ground truth) labels or are part of the HAR and ADL detection model.

In this section we describe our methodology for building an ADL detection model starting from the smartphone sensors as basis and researching which other ambient modalities can improve the ADL classification. Our methodology relies mainly on the accelerometer sensor in a smartphone, but also considers other smartphone modalities, such as android’s built-in step detection, the ambient light sensor, the screen status and the application activity, i.e. whether or not there is an application actively running in the foreground. We adopted a hybrid approach in which we first performed low-level activity detection based on the smartphone accelerometer. The probabilities for each of the low-level activities were then used as features for detecting ADLs. We then gradually incorporated features from other smartphone sensors and monitored their impact on the accuracy for detecting ADLs. Finally, we also incorporated ambient sensors to evaluate how they influenced the ADL detection performance. An overview of all the modalities and algorithms that make up our methodology can be found in Fig. 1. In the following subsections we describe each block in our methodology in more detail.

The low-level activities that we detect are: lying down, (smartphone) on table, running, sitting, standing, and walking. Our approach has two steps: First, in section “Non-intrusively obtaining labels for training the smartphone-based low-level HAR model”, we obtain low-level activity labels to train the model (see section “Empactica wearable HAR model”) and design a threshold-based model to detect when the smartphone is on a table (or other surface, i.e. not on the person) (see section “Phone on table detection”) Next, in section “Low-level HAR”, we use these labels to low-level activity recognition model based on smartphone accelerometer data.

After the low-level activity detection, the smartphone-based ADL detection is detailed in section “ADL detection”. First, we trained an ML model that uses as features only the prediction probabilities of the low-level classes that we obtained from the low-level activity detection model (as described in section “Low-level HAR”). We afterwards added features from other smartphone sensors and subsequently ambient sensors and analyzed the impact on the performance of the ADL detection model.

In this study we re-use data from a public dataset of anonymized data (https://dahcc.idlab.ugent.be/). The original data collection of the DAHCC dataset was approved by the ethical committee of the Faculty of Engineering and Architecture of Ghent University, in accordance with the relevant guidelines and regulations. All participants have signed an informed consent to make their data publicly and anonymously available.

### Non-intrusively obtaining labels for training the smartphone-based low-level HAR model

#### Empactica wearable HAR model

As in the DAHCC dataset^23^ no low-level activity labels are available, the first step was to generate (ground truth) labels for the low-level activities that could be used as features for the ADL detection models. As can be seen in Table 1, there is data available from both smartwatches and smartphones. In previous work^34^, based on another dataset containing annotations of low-level activities performed in the real-world, a boosted trees model was trained on wrist-worn accelerometer data from an Empatica E4 wearable using human-engineered features. This model achieved an overall performance of 90% of balanced accuracy. It is worth noticing that this model used sleeping data as lying down activity, so the results reported in^34^ do not reflect the accuracy of this model for, for example, detecting lying down in a sofa. We therefore obtained labels for the low-level activities performed in the DAHCC dataset by processing the wrist-worn accelerometer data, and using the pre-trained model to make low-level activity predictions on this data. As such, we obtained activity probability predictions every 7.5 s for the following activities: lying down, running, sitting, standing, and walking. Next, we applied label smoothing and per 1-min aggregations of predictions as a post-processing step. The results of this post-processing were our final, per minute, low-level activity labels obtained from the wrist-worn accelerometer.

#### Phone on table detection

It is important to detect when a smartphone is lying stationary on a surface, e.g. a table, as then the accelerometer sensor of the smartphone becomes unreliable for ADL detection. Since the labels for the low-level activities were obtained from a wrist-worn accelerometer, these labels can not be used when the phone is on table. We therefore developed a *Phone On Table* detection module. This module solely uses the smartphone accelerometer and is threshold based. We use a 1 s window and look at the mean value of the Z axis and the standard deviation in all three axes separately. We require that the mean value of the Z axis is greater than 9 or lower than -9 as then the smartphone is positioned screen up or down respectively. We additionally require that the standard deviation is lower than 0.7 in each axis (to be robust for sensor noise). Using these thresholds, we can identify the periods when the smartphone is lying stationary. Next, we combined these periods with the predictions obtained from the wrist-worn activity model by overruling the wrist-worn predictions with the *on table* predictions whenever we detected that the smartphone was stationary. This way we obtained labels for the following classes: lying down, on table, running, sitting, standing, and walking.

### Low-level HAR

**Figure 2:**
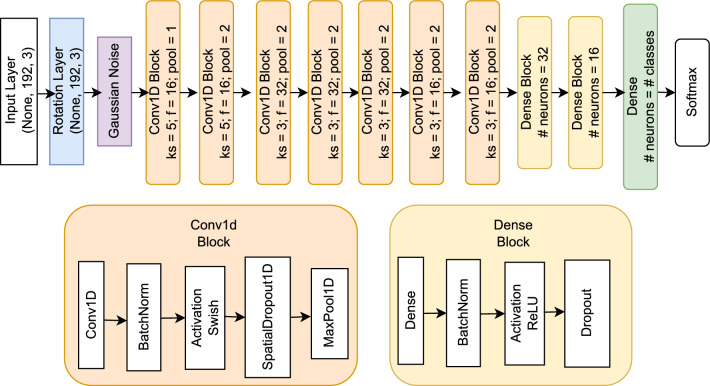
Low-level HAR architecture.

Before building our smartphone-based low-level activity recognition model, we threw away the Running labels obtained from the pre-trained wearable-based model, as we considered this irrelevant for detecting ADLs and only two out of the 31 participants in the dataset had collected running data. We trained a CNN model that can predict the following 5 classes: lying down, on table, sitting, standing, and walking. Based on experience from designing low-level activity detection models based on wrist-worn accelerometer data^29^, a CNN architecture was chosen for its simplicity yet effectiveness for this task. The architecture is shown in Fig. 2.

We used 6 s windows of accelerometer data with 3 s of stride, resulting in an input of length 192 data points. We considered this window length to be long enough to capture the essence of an activity, but not too long risking including more activities in the window. As can be seen in Fig. 2, the architecture consists of 7 convolutional layers with either 16 or 32 filters, kernel sizes of 5 resp. 3, and we apply max pooling. At the end we added two dense layers of 32 resp. 16 neurons, and a softmax layer. The output is a 6 values long vector, each value indicating the probability of the corresponding low-level activity. This architecture has been shown effective by the authors^29^ for detecting similar activities. For data augmentation we incorporated a custom rotation layer that performs small random rotations on the accelerometer data. One can think of this layer as simulating small differences positions of placing / hanging the smartphone (attached not fully straight upwards on the shirt, tilting a little forward or backward due to looser shirt, etc.). This makes our model more robust and adequate for real-world scenarios. We also added Gaussian noise on the input data, and used spatial dropout and normal dropout for regularization.

For evaluation, we kept 10 participants as a holdout set. The other 21 participants were used for tuning the hyper-parameters using 4-fold group cross-validation where each fold consisted of train data from 15/16 participants and validation data from 6/5 participants. Doing this was more time-efficient for hyperparameter tuning than 5-fold, and resulted in a better divided train/validation/test split. For optimizing the hyper-parameters we used the hyperopt package^35^. Results of the model are discussed in section “Low-level HAR detection model”.

### ADL detection

The ADLs that we considered are: preparing drink, preparing meal, toileting, eating (including drinking), using the computer, watching TV, walking, showering, organizing, using the smartphone, room transition, and other (all the other activities that are not one of the previous ones). These ADLs were chosen in collaboration with healthcare experts of the imec.ICON PROTEGO^36^ consortium as these activities are considered to be core activities of the daily lives of elderly that are frequently linked to the occurrence of alarming situations, e.g., a fall, or lack of nutrition, hydration or movement. As such, they are a crucial part of monitoring human behavior, and thus their accurate detection is of great importance. Below, we first discuss which additional smartphone sensors (section “Phone sensors and features”) and ambient sensors (section “Ambient sensors and features”) were selected to combine with the low-level activity detection to perform ADL detection from all the data that was collected within the DAHCC dataset. Next, we discuss how the ADL labels were provided by the participants and how they were cleaned up to enable better training of the ADL detection model (section “Label handling”). Finally, we detail the architecture of the ML model (section “ML model architecture”) and the accompanying hyperparameter optimization (section “Hyper-parameters optimization and evaluation”).

#### Phone sensors and features

Every smartphone is equipped with a variety of sensors. In this work we included, as already stated, the motion sensor, i.e. accelerometer, as basis. We used the accelerometer indirectly, by first using the raw accelerometer data to make predictions for the low-level activities. The probabilities for these low-level activities are then used as features for the ADL detection. We also considered the following additional smartphone sensors: (i) the Android built-in *step detector*, from which we derive the number of steps taken in the sample time window as feature; (ii) the *ambient light* sensor that measures light intensity in lux, sampled at 1 Hz, from which we use the window mean value as feature; and (iii) we also created a *phone usage* feature (whether or not a person is using the smartphone).

We detect the start and end time of active interaction with the smartphone based on two features, i.e. the *screen status* (on, off, locked, unlocked) and whether an application has been opened. During data collection we logged an entry every time a new app came to the foreground on the smartphone. An entry consists of the app name and the timestamp at which it was opened. This resulted in a list of apps and the corresponding opening times. From this list of opened applications, we first removed applications that we consider to be system utilities, such as the keyboard appearing within an application. The list of system utilities can be found in the Supplementary Information. Applications can disappear from the foreground for two reasons, i.e. the user switches to another application or the user stops using the smartphone. To capture the latter, we insert *Off* entries in the filtered list of opened applications at the timestamps that the app stopped being in foreground, either because the screen of the smartphone got locked or the phone screen was turned off. We then sorted the list based on the timestamps in ascending order. Next, we added to each entry, the timestamp of the successor app, or in certain cases the timestamp of the screen going *Off*, which represents the end time of using this app. This results in a list of apps with their respective start and end usage times. Some of these entries are *Off* entries, which in reality is not an app but the period in which the screen was off or locked. We removed these entries from the list, as these are moments in which no app was in the foreground and therefore the smartphone was not used by the person. The final list included entries which correspond to periods in which the person is actively using the apps and smartphone. The feature we generated from this list is a binary feature indicating whether the person is using the smartphone at a given moment.

To summarize, we considered three additional smartphone features next to the accelerometer: number of steps taken, mean light intensity, and whether or not the person was actively using the smartphone in the given window.

#### Ambient sensors and features

Even though there are many sensors available in the DAHCC dataset, in this work we considered only a small subset. We chose the subset of sensors based on few grounds: (i) relevancy to the ADLs we were targeting, and (ii) quality and reliability of the sensor data. We also looked at cost and ease of installment of the sensors in the home to further refine the selection.

One of the ambient sensors we selected was the *AQURA location sensor*. Room level localisation is one of the most valuable sensors when it comes to elderly healthcare and monitoring, not just for activity recognition but also for emergency cases. As room level localisation is very relevant for ADL detection, we decided to incorporate it in our research. The sensor is a proximity-based indoor localisation^37^ solution that combines beacons and a sensor that is worn on the body that sends a value every time the sensor detects a different location. From this localisation data we extract a feature that indicates in which room the person is in the given time window.

An *EnOcean fridge contact sensor* was used to detect when the fridge was opened. This sensor is very cheap and easy to install, while providing valuable information for ADL detection. From this data, we generate a feature equal to the number of seconds since the fridge was last opened. As previously mentioned, ADLs have a temporal dependency, which is one of the reasons why we chose for this feature instead of only instantaneous and binary open/closed feature.

Next, a *Velbus power consumption sensor* was used to measure the power consumption of major appliances. In this work we monitored the power consumption of five appliances that are used a lot during the ADLs we are trying to detect: cooking plate, cooking hood, electric water kettle, coffee machine, and TV. For each of the appliances we calculated a feature that represents the number of seconds since the appliance was used for the last time. Similarly to the reasoning for the fridge feature, also here, we consider this feature more informative than a simple binary on/off feature. The features were normalized over a 12h period. The Velbus power consumption sensor is nor easy, nor cheap to install, but in this work we used it only as if it were a binary switch (on/off) sensor. So the Velbus sensor can be replaced with a cheap and easy to install switch sensor without losing adequacy for our methodology.

Finally, a *light sensor* was used to measure the light intensity in the two toilets and the bathroom. In the toilets the lights are motion triggered and turn on when a person enters the toilet. The bathroom did not have natural lighting so a person always had to turn on the light when they were in the bathroom. The feature we calculated from these sensors is a binary feature that indicates whether the light is on or off in the given room. In our case this can be interpreted as whether or not the person is in the given room. We did not consider the light sensors in other rooms as they did not immediately correlate to whether the person was in that room or not. We explored the light sensor based feature, primarily, to check if these light sensors would be an adequate alternative for the AQURA (localisation) sensor.

#### Label handling

The ground truth ADL labels in the DAHCC^23^ dataset are obtained through self-reporting. Participants logged their ADL through a smartphone application. As the dataset aimed to reflect a real-world situation as closely as possible, participants were given very few labeling instructions. Mainly they were asked to indicate the start and the end time of the period in which they performed the ADL(s) and provide the label(s) thereof. As a consequence, a variety of labeling strategies have emerged, varying according to individual preferences. Some participants tried to provide single labels wherever possible, even if the activity was very short, e.g., room transition. Others would group several activities in a single input and thus same time period, e.g. preparing food and going to the toilet.

Even though it is possible to perform two or more activities at the same time, such as eating and watching TV, for other activities it is clear that they can only happen one after the other. For example, some people labeled in a single input working and room transition, or room transition and toileting. It is clear that these activities can not happen concurrently, but this way there are no specific times of where the first activity ended and the second started. Therefore, in the cases where room transition appeared as first or last activity, we allocated 30 s to the room transition activity (the first resp. last 30 s of the provided period), and reserved the remaining time for the other labeled activities in this time period. Other situations in which two or more labels were provided are, for example, preparing meal and toileting. It can happen that the person starts preparing a meal, e.g., puts a pot of water on the cooking top, and goes to the toilet while waiting for it to boil. As in this case we did not have the necessary information on when exactly the toileting activity took place, we decided to consider the activity that the person actively engaged in as the label, e.g., in this case toileting. The same principle was applied for other examples of time periods that received multiple simultaneous labels, such as using a computer and toileting, using a computer and organizing, watching TV and toileting, and eating and toileting.

People also engaged in, and thus also labeled, other activities which are irrelevant for the ADL detection use case. For these, we assigned the *Other* class that includes all activities beyond the 11 selected ADL activities. Finally, we also had periods of data without any label. We still used this data in the training process, in a way such that the predictions of these periods do not have effect on the training loss. This is explained in more detail in the following section “ML model architecture”.

#### ML model architecture

**Figure 3:**
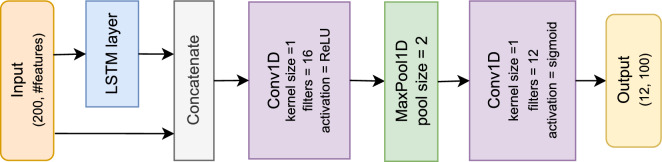
Schematic view of our architecture.

The architecture we have chosen for the ADL detection is a Long Short Term Memory (LSTM) layer followed by two convolutional layers. LSTMs^38^ have a chain like structure in which the repeating module consists of four neural network layers (three sigmoid layers and one tanh layer). The core idea of an LSTM is that the cell state and the four neural network layers interact to decide what information will be forgotten, what new information will be stored, and what the output will be. This way, LSTMs address the long-term dependency issue that other networks, such as Recurrent Neural Networks (RNNs) had. LSTMs are therefore adequate for our challenge as ADLs have temporal dependencies and (past) states of the situation are important for detecting transitions and hence activities. Figure 3 gives a schematic overview of our ADL detection architecture.

We have decided to work with windows of 10 min, with 2 min stride. We believe that 10 min is long enough to capture several ADLs within the window to learn relationships between them: such as eating after having cooked, even if one went to the toilet in between. For the same reason we chose 2 min stride to have sufficient opportunities to capture related activities in a single window. As there could be more than one ADL being performed within those 10 min, and, additionally, also one or more ADL concurrently, we formulated the problem as a multi-label classification. We therefore did not use a fully connected layer, but 12 filters instead in the last convolutional layer, where 12 is the number of activities we are detecting, i.e. the classification classes. We then applied a sigmoid activation function on the output of the last convolutional network, and performed a binary classification for each of the activities.

As such, the input data to our network consists each time of a window of 10 min long. This window contains a data point every 3 s, and thus has a length of 200 data points in the time dimension. To study which (combination of) sensor modalities are required to detect (specific) ADLs, we experimented with the following different feature vectors to represent the data points every 3 s:

- probabilities of low-level activities only,
- probabilities of low-level activities, phone use and steps,
- probabilities of low-level activities and features of appliances usage,
- probabilities of low-level activities and all other phone features,
- probabilities of low-level activities and location in the home,
- all features, i.e. probabilities of low-level activities, plus all other phone features, plus ambient sensors and location,
- all features except location, and finally,
- features from ambient sensors only.

The LSTM layer returns the full sequence, for every time point, rather than only the last sequence. We therefore made a skip connection and concatenated the output with the original input fed to the first convolutional layer. In the first convolutional layer, we applied 16 filters of kernel size 1, and ReLu as activation function. We also applied a max pooling of size 2 as this reduces the time dimension to 100. The latter is used as the input to the second convolutional layer where we applied 12 filters (equal to the number of activities we want to detect) of kernel size 1, no pooling and a sigmoid activation function, so that we can perform binary classification over the 12 activities.

As this is a densely-labeled multi-label classification problem, we chose the loss function to be the mean of the 12 balanced binary cross-entropy losses corresponding to the 12 classes, weighted along the time dimension to reflect the presence of labels.

The weights along the time dimension were set to zero to accommodate for the unlabeled time points. This way we allow the LSTM model, which needs continuous chunks of data, to look at the sensor data / features, but we do not consider predictions for the unlabeled time points in the loss function.

As the activities are of varying length and probability of occurrence throughout a normal day, this resulted in a highly imbalanced dataset. For example, using computer is an activity that can last for hours, whereas room transition is normally always very short. As a result, there are many more time points labeled as *not room transition* than time points labeled as *room transition*. We therefore used binary class weights (class being positive or negative here) for each activity to address this imbalance issue.

#### Hyper-parameters optimization and evaluation

For hyper-parameter optimization and evaluation we adopted the same approach and data splits as in section “Low-level HAR”. We kept 10 participants as a holdout set, and the other 21 participants were used for hyper-parameter tuning using a 4-fold cross-validation. The following hyper-parameters were optimized: learning rate, number of LSTM units, dropout rate, Gaussian noise, and l1 and l2 regularization. We fixed the number of epochs to 350 for all experiments, as preliminary testing showed that models converged by this number. The batch size was fixed to 1024.

As we already mentioned in section “Label handling”, the provided ADL labels are not 100% reliable. As such, we adopted several ways to evaluate our methodology: (a) binary classification metrics for each activity separately, such as logloss, balanced accuracy, F1-micro, F1-macro, true positive rate (TPR), and true negative rate (TNR), (b) binary confusion matrices for each activity, and (c) a confusion matrix that includes all the activities together.

The definition of precision and recall are given in Eqs. (1) and (2) respectively.

1 $$\begin{aligned} \text {Precision} = \frac{\text {TP}}{\text {TP}+\text {FP}} \end{aligned}$$

2 $$\begin{aligned} \text {Recall} = \frac{\text {TP}}{\text {TP}+\text {FN}} \end{aligned}$$

where TP are the true positives, FP the false positives and FN are the false negatives, **F1-micro** is a measure of the overall accuracy, where the precision and recall of each class is calculated globally (across all classes) rather than independently, in Eq. (3)

3 $$\begin{aligned} F1_{micro} = 2 \frac{(\text {Precision} \cdot \text {Recall})}{(\text {Precision} + \text {Recall})} \end{aligned}$$

**F1-macro** measures the average accuracy, where the precision and recall of each class are calculated independently and then averaged together, as shown in Eq. (4)

4 $$\begin{aligned} \text {F1}_{macro} = \frac{1}{C} \sum _{i=1}^{C} 2 \frac{(\text {Precision}_i \cdot \text {Recall}_i)}{(\text {Precision}_i + \text {Recall}_i)} \end{aligned}$$

where *C* is the number of classes. And finally, **balanced accuracy** is defined as the average of the recall for each class as shown in Eq. (5), while **LogLoss** shows how close a prediction probability comes to the actual/corresponding true value as defined in Eq. (6):

5 $$\begin{aligned} \text {BalancedAccuracy} = \frac{1}{C} \sum _{i=1}^{C} \text {Recall}_i \end{aligned}$$

6 $$\begin{aligned} \text {logloss} = -\frac{1}{N} \sum _{i=1}^{N} \sum _{j=1}^{C} y_{i,j} \log (p_{i,j}) \end{aligned}$$

where *C* is the number of classes, *N* is the number of samples, $y_{i,j}$ is the true label of sample *i* for class *j*, and $p_{i,j}$ is the predicted probability of sample *i* for class *j*.

Besides calculating the binary classification metrics for each activity separately, we also provide a full confusion matrix that includes all the activities together. Since we used multi-label classification, we cannot construct a confusion matrix out of the box, as every time point can have multiple labels.

**Figure 4:**
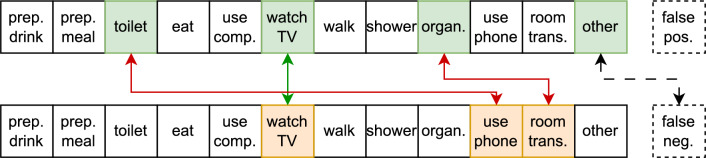
Schematic visualisation of how we “flatten” the labels and predictions. A case where there are more labeled ADLs than predicted.

**Figure 5:**
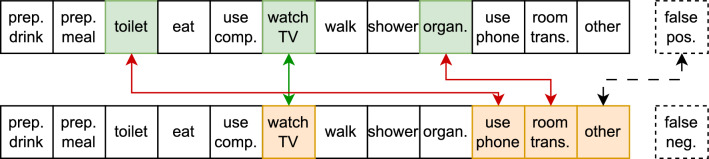
Schematic visualisation of how we “flatten” the labels and predictions. A case where there are more predicted ADLs than labels.

To overcome this, for every prediction of the model, consisting of one or more predicted ADLs, and having one or morelabels, we perform a prediction-label matching algorithm to produce a “flattened” list of single-prediction single-label pairs by following three steps:

- Step 1: If an ADL is present in both the set of predictions and the set of labels, they are matched and added to the list of single-prediction single-label pairs. An example of this scenario is the activity *WatchingTV* in the example shown in Figs. 4 and 5.
- Step 2: As long as there are unmatched ADLs left in both the predicted set and the label set, they are matched in arbitrary order (and thus will appear as confused ADLs in the confusion matrix). An example of this scenario is shown in Figs. 4 and 5 where the labeled ADLs *Toileting* and *Organizing* are matched with wrongly predicted ADLs *Using Phone* and *Room Transition*. As we are unable to determine which identified ADL corresponds to each labeled ADL, we consistently include them in a random sequence. This introduces some randomness in the confusion matrix, but we empirically noted that this only rarely happens so we proceeded with this approach.
- Step 3: Finally, if there are unmatched predictions or labels left, two things can happen. In case of a label without a prediction, a false negative pair is generated for each of the remaining labels. This is shown in Fig. 4 where the labeled ADL *Other* is matched with a “False Negative”. In case of a prediction without a label, a false positive pair is generated for each of the remaining predictions. This is depicted in Fig. 5 where the wrongly detected ADL *Other* is matched with a “False Positive”.

As such, the amount of single-label single-prediction pairs is the maximum of the amount of predictions and the amount of labels, leading to a rather underestimation of performance.

We repeated the above procedure for each time point in the test data. This results in a confusion matrix with all labeled activities plus a row with label “false positive” and a column “false negative”. This is useful to inspect which activities were often predicted but didn’t happen, and which ones were missed.

## Results

In this section we provide the results of the performed experiments. In section “Low-level HAR detection model”, we first present the results of the CNN model that performs the low-level HAR, and then, in section “ADL detection models” we present the results of our LSTM models that detect the ADL, trained with different subset of features as discussed in section “ML model architecture”.

### Low-level HAR detection model

**Table 2:** Results of the low-level HAR model on the hold-out set. Mean and std across the 10 participants in the holdout set for the different performance metrics.

	LogLoss	Balanced accuracy	F1-macro	F1-micro
	$0.62 \;\pm \;0.17$	$0.67 \;\pm \;0.12$	$0.52 \;\pm \;0.08$	$0.74 \;\pm \;0.08$

**Figure 6:**
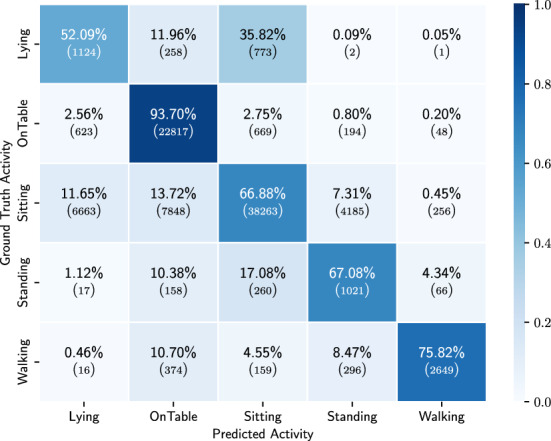
Confusion matrix of the results of the low-level HAR model on the hold-out set.

In Table 2 we present the results of the CNN for detecting the low-level HAR. For each performance metric we show the mean value and the standard deviation (std), obtained across the 10 participants in the holdout set. In Fig. 6 we show the confusion matrix obtained on all 10 participants together. The average balanced accuracy is 67%. Lying is the activity that is least accurately predicted, often mistaken for sitting. The best detected activity is phone on table, followed by the walking activity.

### ADL detection models

In this section we report the results of the ADL detection models trained on different sensor subsets. We present results for two different combinations, the results of other combinations can be found in the Supplementary Information. These combinations are: only low-level probabilities, low-level probabilities and location in the home. We chose these combinations because they allow for a discussion on the trade-off between performance versus cost and overhead of including more (ambient) sensors. Finally we present results from a baseline model that does not include LSTM layer, to show the relevance of including LSTM layer in the architecture.

#### Only probabilities

**Table 3:** Results of the ADL detection model, trained on only probabilities of the detected low-level activities. Mean and std across the 10 participants in the holdout set for the different performance metrics are shown.

	LogLoss	Balanced accuracy	F1-macro	F1-micro	TPR	TNR
Preparing drink	$0.45 \;\pm \;0.07$	$0.73 \;\pm \;0.16$	$0.51 \;\pm \;0.03$	$0.86 \;\pm \;0.03$	$0.59 \;\pm \;0.34$	$0.87 \;\pm \;0.04$
Preparing meal	$0.37 \;\pm \;0.13$	$0.77 \;\pm \;0.14$	$0.65 \;\pm \;0.07$	$0.86 \;\pm \;0.04$	$0.65 \;\pm \;0.30$	$0.88 \;\pm \;0.05$
Toileting	$0.48 \;\pm \;0.14$	$0.80 \;\pm \;0.10$	$0.57 \;\pm \;0.07$	$0.83 \;\pm \;0.04$	$0.77 \;\pm \;0.20$	$0.83 \;\pm \;0.04$
Eating	$0.84 \;\pm \;0.10$	$0.55 \;\pm \;0.10$	$0.44 \;\pm \;0.10$	$0.55 \;\pm \;0.15$	$0.56 \;\pm \;0.15$	$0.55 \;\pm \;0.17$
Using computer	$0.69 \;\pm \;0.20$	$0.66 \;\pm \;0.10$	$0.62 \;\pm \;0.14$	$0.65 \;\pm \;0.09$	$0.68 \;\pm \;0.22$	$0.64 \;\pm \;0.10$
Watching tv	$0.89 \;\pm \;0.29$	$0.68 \;\pm \;0.13$	$0.54 \;\pm \;0.11$	$0.68 \;\pm \;0.15$	$0.67 \;\pm \;0.22$	$0.69 \;\pm \;0.16$
Walking	$0.19 \;\pm \;0.12$	$0.91 \;\pm \;0.14$	$0.82 \;\pm \;0.15$	$0.96 \;\pm \;0.03$	$0.85 \;\pm \;0.29$	$0.97 \;\pm \;0.03$
Showering	$0.28 \;\pm \;0.31$	$0.90 \;\pm \;0.10$	$0.61 \;\pm \;0.17$	$0.85 \;\pm \;0.18$	$0.87 \;\pm \;0.29$	$0.85 \;\pm \;0.18$
Organizing	$0.63 \;\pm \;0.06$	$0.76 \;\pm \;0.16$	$0.54 \;\pm \;0.05$	$0.82 \;\pm \;0.06$	$0.70 \;\pm \;0.36$	$0.82 \;\pm \;0.06$
Using phone	$0.74 \;\pm \;0.29$	$0.67 \;\pm \;0.10$	$0.49 \;\pm \;0.14$	$0.62 \;\pm \;0.19$	$0.72 \;\pm \;0.27$	$0.62 \;\pm \;0.23$
Room transition	$0.73 \;\pm \;0.07$	$0.61 \;\pm \;0.16$	$0.45 \;\pm \;0.05$	$0.64 \;\pm \;0.07$	$0.58 \;\pm \;0.36$	$0.64 \;\pm \;0.12$
Other	$0.71 \;\pm \;0.20$	$0.62 \;\pm \;0.11$	$0.50 \;\pm \;0.17$	$0.58 \;\pm \;0.21$	$0.68 \;\pm \;0.20$	$0.56 \;\pm \;0.26$
$Mean \;\pm \;std$	$0.58 \;\pm \;0.22$	$0.72 \;\pm \;0.11$	$0.56 \;\pm \;0.10$	$0.74 \;\pm \;0.14$	$0.69 \;\pm \;0.10$	$0.74 \;\pm \;0.14$

**Figure 7:**
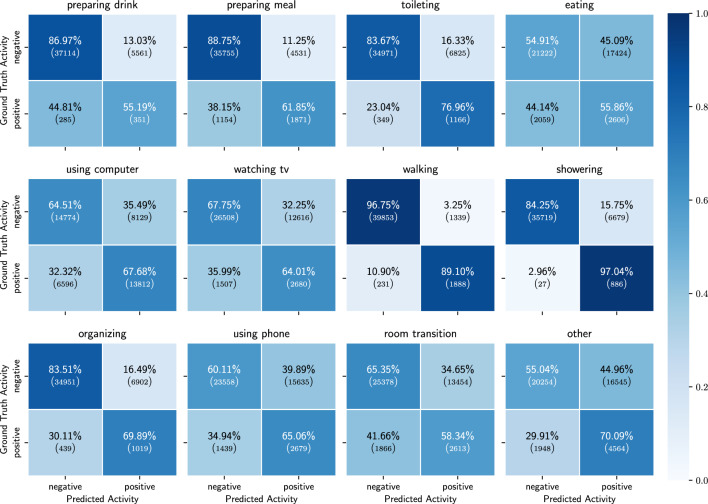
Binary confusion matrices of the results of the ADL detection model, trained on only probabilities of the detected low-level activities.

**Figure 8:**
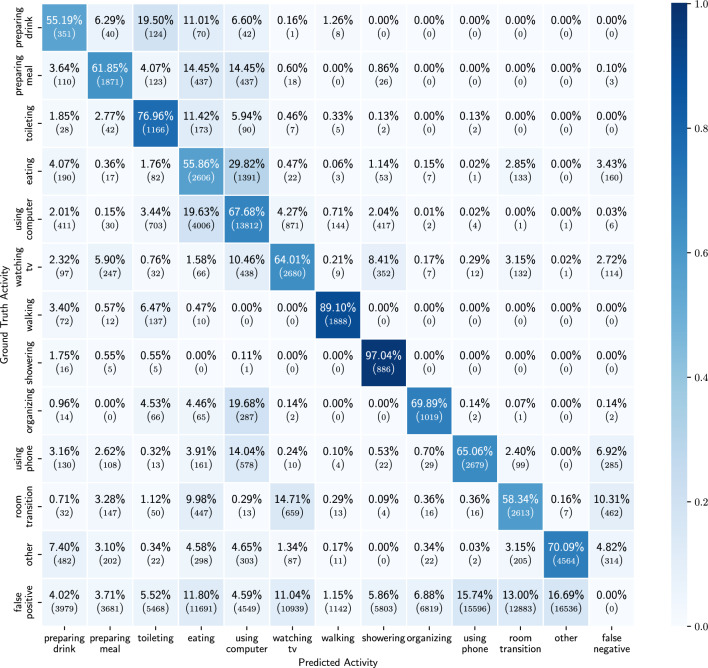
Confusion matrix of the results of the ADL detection model, trained on only probabilities of the detected low-level activities.

In this section we show the results obtained when using only the probabilities of the detected low-level activities as features.  Table 3 shows the mean and std of the performance metrics for each activity across the 10 participants in the holdout set. For all of the activities we observe an average balanced accuracy of 55% or more. The best distinguished activities are walking, showering and toileting, while eating is the activity for which the model performs the worst. Figure 7 shows the confusion matrices for all activities over all the 10 participants in the holdout set. From these figures we can observe that for the activities eating, using phone and room transition, the model is often confused. However, preparing meal, using computer and watching TV are all activities for which it is not straightforward that they would be detected solely based on low-level activity features and yet they are detected more than 60% of the time. Figure 8 shows the full confusion matrix after processing the predictions and labels as described in section “Hyper-parameters optimization and evaluation”. We can see that eating and using computer are often mistaken for each other. This is to some extent to be expected, as both activities are most of the time performed while sitting on a chair with a table in front.

#### Probabilities and location

**Table 4:** Results of the ADL detection model, trained on probabilities of low-level activities and location. Mean and std across the 10 participants in the holdout set for the different performance metrics is shown.

	LogLoss	Balanced accuracy	F1-macro	F1-micro	TPR	TNR
Preparing drink	$0.50 \;\pm \;0.16$	$0.84 \;\pm \;0.09$	$0.51 \;\pm \;0.04$	$0.81 \;\pm \;0.08$	$0.86 \;\pm \;0.15$	$0.81 \;\pm \;0.08$
Preparing meal	$0.34 \;\pm \;0.16$	$0.85 \;\pm \;0.11$	$0.71 \;\pm \;0.09$	$0.87 \;\pm \;0.07$	$0.81 \;\pm \;0.21$	$0.88 \;\pm \;0.07$
Toileting	$0.22 \;\pm \;0.10$	$0.94 \;\pm \;0.04$	$0.74 \;\pm \;0.09$	$0.94 \;\pm \;0.03$	$0.94 \;\pm \;0.05$	$0.94 \;\pm \;0.03$
Eating	$0.72 \;\pm \;0.17$	$0.62 \;\pm \;0.11$	$0.48 \;\pm \;0.14$	$0.57 \;\pm \;0.16$	$0.69 \;\pm \;0.15$	$0.56 \;\pm \;0.17$
Using computer	$0.42 \;\pm \;0.13$	$0.80 \;\pm \;0.08$	$0.77 \;\pm \;0.12$	$0.83 \;\pm \;0.07$	$0.82 \;\pm \;0.18$	$0.78 \;\pm \;0.09$
Watching tv	$0.49 \;\pm \;0.15$	$0.76 \;\pm \;0.12$	$0.74 \;\pm \;0.12$	$0.89 \;\pm \;0.06$	$0.60 \;\pm \;0.21$	$0.93 \;\pm \;0.05$
Walking	$0.10 \;\pm \;0.12$	$0.93 \;\pm \;0.14$	$0.85 \;\pm \;0.15$	$0.97 \;\pm \;0.03$	$0.88 \;\pm \;0.28$	$0.98 \;\pm \;0.03$
Showering	$0.09 \;\pm \;0.06$	$0.98 \;\pm \;0.02$	$0.78 \;\pm \;0.14$	$0.98 \;\pm \;0.02$	$0.88 \;\pm \;0.30$	$0.97 \;\pm \;0.02$
Organizing	$0.63 \;\pm \;0.17$	$0.74 \;\pm \;0.09$	$0.47 \;\pm \;0.10$	$0.66 \;\pm \;0.14$	$0.84 \;\pm \;0.18$	$0.65 \;\pm \;0.15$
Using phone	$0.73 \;\pm \;0.22$	$0.66 \;\pm \;0.11$	$0.51 \;\pm \;0.14$	$0.67 \;\pm \;0.20$	$0.64 \;\pm \;0.29$	$0.68 \;\pm \;0.24$
Room transition	$0.74 \;\pm \;0.11$	$0.65 \;\pm \;0.11$	$0.46 \;\pm \;0.10$	$0.62 \;\pm \;0.13$	$0.67 \;\pm \;0.27$	$0.64 \;\pm \;0.14$
Other	$0.70 \;\pm \;0.20$	$0.65 \;\pm \;0.09$	$0.58 \;\pm \;0.11$	$0.68 \;\pm \;0.14$	$0.61 \;\pm \;0.14$	$0.69 \;\pm \;0.17$
$Mean \;\pm \;std$	$0.47 \;\pm \;0.24$	$0.79 \;\pm \;0.13$	$0.63 \;\pm \;0.14$	$0.79 \;\pm \;0.14$	$0.77 \;\pm \;0.12$	$0.79 \;\pm \;0.15$

**Figure 9:**
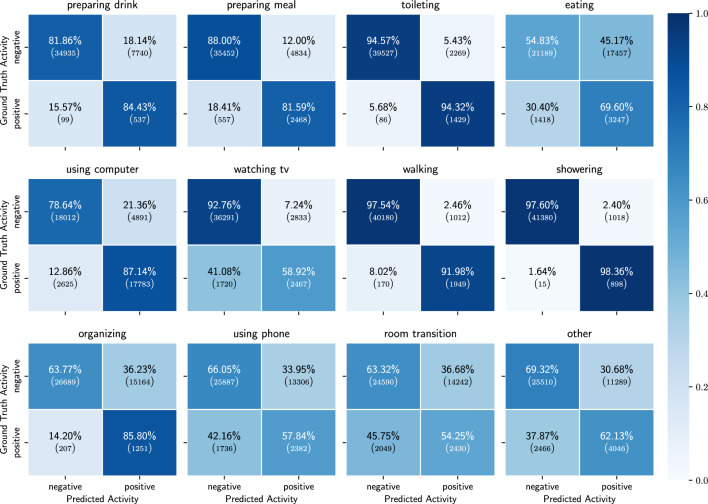
Binary confusion matrices of the results of the ADL detection model, trained on probabilities of low-level activities and location.

**Figure 10:**
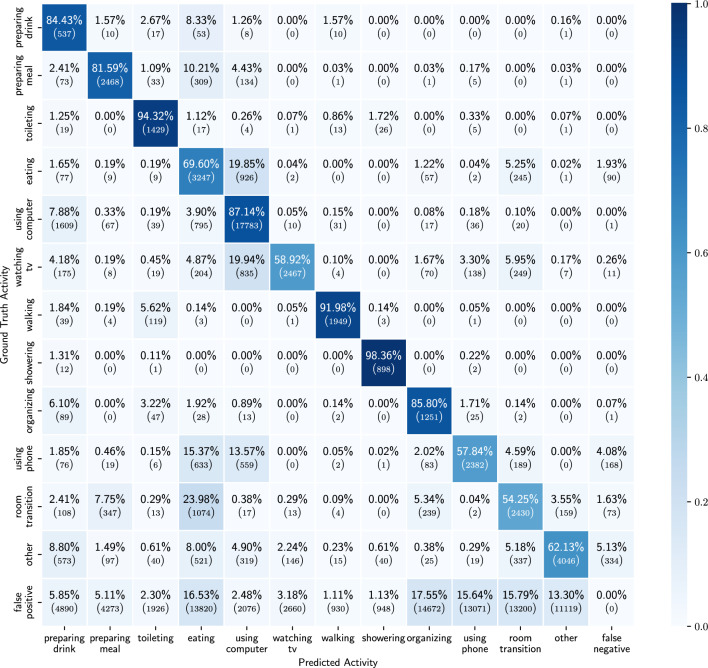
Confusion matrix of the results of the ADL detection model, trained on probabilities of low-level activities and location.

We obtain the best results when using the probabilities of the low-level activities combined with the location sensor as input source. In Table 4 we see that for all of the activities we achieve a balanced accuracy of more than 60% and in Fig. 9 we can see that the TP ratio is above 50% for all activities as well. One noticeable degradation in the results is the TP rate of watching TV. Compared to the results when using all sensors, we see a decrease in TP rate of around 8%. This is explained by the fact that we do not have explicit information from the TV energy usage. Interesting to notice are the results of preparing drink and preparing meal compared to the ones from using only probabilities for the low-level activities. As both take place in the kitchen one would expect similar results as when using only probabilities. However, this is not the case and we obtain better results when we include the location information. This may indicate that there is a certain location pattern prior to going to the kitchen for preparing drink or preparing meal (Table 5).

### Baseline model

**Table 5:** Results of the baseline ADL detection model, trained on probabilities of low-level activities and location. Mean and std across the 10 participants in the holdout set for the different performance metrics is shown.

	LogLoss	Balanced Accuracy	F1-macro	F1-micro	TPR	TNR
Preparing drink	$0.53 \;\pm \;0.08$	$0.81 \;\pm \;0.08$	$0.51 \;\pm \;0.03$	$0.83 \;\pm \;0.06$	$0.80 \;\pm \;0.15$	$0.83 \;\pm \;0.06$
Preparing meal	$0.35 \;\pm \;0.10$	$0.84 \;\pm \;0.11$	$0.69 \;\pm \;0.08$	$0.86 \;\pm \;0.06$	$0.81 \;\pm \;0.22$	$0.87 \;\pm \;0.05$
Toileting	$0.22 \;\pm \;0.07$	$0.90 \;\pm \;0.02$	$0.72 \;\pm \;0.08$	$0.94 \;\pm \;0.02$	$0.87 \;\pm \;0.05$	$0.94 \;\pm \;0.02$
Eating	$0.74 \;\pm \;0.09$	$0.58 \;\pm \;0.16$	$0.47 \;\pm \;0.12$	$0.57 \;\pm \;0.14$	$0.61 \;\pm \;0.34$	$0.56 \;\pm \;0.16$
Using computer	$0.54 \;\pm \;0.17$	$0.78 \;\pm \;0.05$	$0.73 \;\pm \;0.14$	$0.77 \;\pm \;0.09$	$0.93 \;\pm \;0.08$	$0.63 \;\pm \;0.09$
Watching tv	$0.61 \;\pm \;0.09$	$0.71 \;\pm \;0.12$	$0.66 \;\pm \;0.14$	$0.83 \;\pm \;0.10$	$0.57 \;\pm \;0.22$	$0.86 \;\pm \;0.11$
Walking	$0.11 \;\pm \;0.12$	$0.95 \;\pm \;0.06$	$0.85 \;\pm \;0.13$	$0.97 \;\pm \;0.03$	$0.94 \;\pm \;0.11$	$0.97 \;\pm \;0.03$
Showering	$0.10 \;\pm \;0.06$	$0.97 \;\pm \;0.03$	$0.79 \;\pm \;0.15$	$0.97 \;\pm \;0.02$	$0.87 \;\pm \;0.29$	$0.97 \;\pm \;0.02$
Organizing	$0.66 \;\pm \;0.09$	$0.77 \;\pm \;0.07$	$0.51 \;\pm \;0.10$	$0.73 \;\pm \;0.12$	$0.81 \;\pm \;0.15$	$0.73 \;\pm \;0.13$
Using phone	$0.73 \;\pm \;0.15$	$0.68 \;\pm \;0.13$	$0.52 \;\pm \;0.10$	$0.71 \;\pm \;0.18$	$0.63 \;\pm \;0.35$	$0.74 \;\pm \;0.23$
Room transition	$0.74 \;\pm \;0.05$	$0.57 \;\pm \;0.10$	$0.49 \;\pm \;0.04$	$0.75 \;\pm \;0.10$	$0.36 \;\pm \;0.28$	$0.78 \;\pm \;0.14$
Other	$0.64 \;\pm \;0.15$	$0.64 \;\pm \;0.08$	$0.61 \;\pm \;0.08$	$0.75 \;\pm \;0.09$	$0.47 \;\pm \;0.11$	$0.80 \;\pm \;0.10$
$Mean \;\pm \;std$	$0.50 \;\pm \;0.24$	$0.77 \;\pm \;0.13$	$0.63 \;\pm \;0.13$	$0.81 \;\pm \;0.12$	$0.72 \;\pm \;0.19$	$0.81 \;\pm \;0.13$

**Figure 11:**
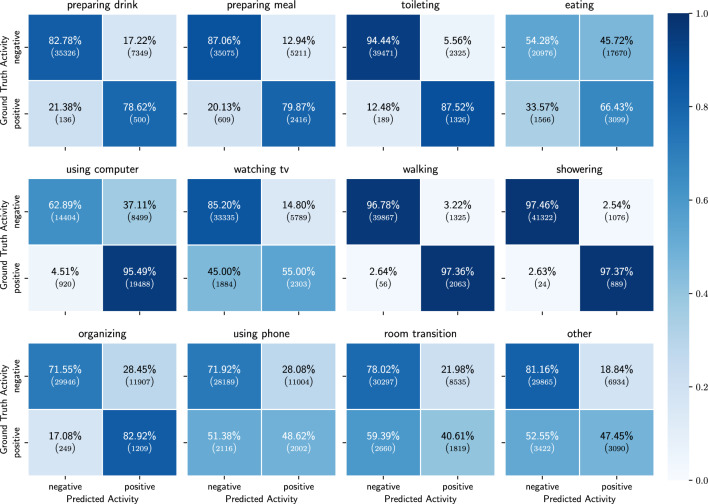
Binary confusion matrices of the results of the baseline ADL detection model, trained on probabilities of low-level activities and location.

**Figure 12:**
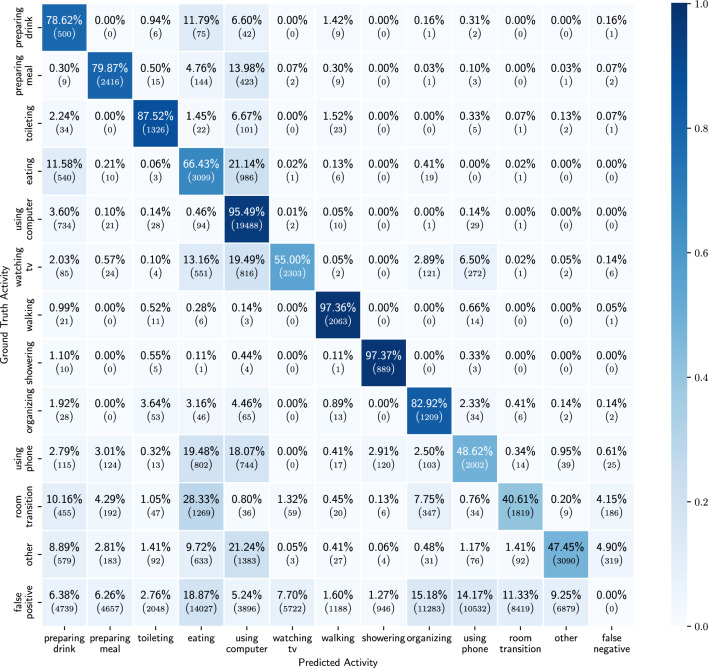
Confusion matrix of the results of the baseline ADL detection model, trained on probabilities of low-level activities and location.

To test the added value of LSTMs and their ability to discover temporal patterns, we trained a model without the LSTM layer. This results in an architecture with two convolutional layers each with kernel size one. Such a model can thus not extract any temporal patterns. The model was tested for the most desirable combination of features, as shown by the previous section, namely low-level activities probabilities and location data. We can see that the results are considerably worse than the results from this model’s LSTM counter part (Figs. 10, 11, 12).

**Table 6:** Balanced accuracy for each of the two presented combinations and the baseline model. Mean and std across the activities for the different performance metrics.

	Only probabilities	Probabilities and location	Baseline probabilities and location
Preparing drink	$0.73 \;\pm \;0.16$	${\textbf {0.84}} \;\pm \;0.09$	$0.81 \;\pm \;0.08$
Preparing meal	$0.77 \;\pm \;0.14$	${\textbf {0.85}} \;\pm \;0.11$	$0.84 \;\pm \;0.11$
Toileting	$0.80 \;\pm \;0.10$	${\textbf {0.94}} \;\pm \;0.04$	$0.90 \;\pm \;0.02$
Eating	$0.55 \;\pm \;0.10$	${\textbf {0.62}} \;\pm \;0.11$	$0.58 \;\pm \;0.16$
Using computer	$0.66 \;\pm \;0.10$	${\textbf {0.80}} \;\pm \;0.08$	$0.78 \;\pm \;0.05$
Watching tv	$0.68 \;\pm \;0.13$	${\textbf {0.76}} \;\pm \;0.12$	$0.71 \;\pm \;0.12$
Walking	$0.91 \;\pm \;0.14$	$0.93 \;\pm \;0.14$	${\textbf {0.95}} \;\pm \;0.06$
Showering	$0.90 \;\pm \;0.10$	${\textbf {0.98}} \;\pm \;0.02$	$0.97 \;\pm \;0.03$
Organizing	$0.76 \;\pm \;0.16$	$0.74 \;\pm \;0.09$	${\textbf {0.77}} \;\pm \;0.07$
Using phone	$0.67 \;\pm \;0.10$	$0.66 \;\pm \;0.11$	${\textbf {0.68}} \;\pm \;0.13$
Room transition	$0.61 \;\pm \;0.16$	${\textbf {0.65}} \;\pm \;0.11$	$0.57 \;\pm \;0.10$
Other	$0.62 \;\pm \;0.11$	${\textbf {0.65}} \;\pm \;0.09$	$0.64 \;\pm \;0.08$
$Mean \;\pm \;std$	$0.72 \;\pm \;0.11$	$0.79 \;\pm \;0.13$	$0.77 \;\pm \;0.13$

Highest result for each activity is indicated in bold.

Table 6 shows the balanced accuracy for each of the three models next to each other. From this table we can see that incorporating location information improves the results. Additionally, we see that for all but three activities the LSTM model outperforms the baseline model.

## Discussion

The main goal of this work was to research the potential of smartphones as a device with all its sensors for ADL recognition. We additionally researched the potential of combining the data from the phone with ambient sensors that can be installed in the home. In this section we will discuss our contributions, limitations and future work.

### Contributions

In this work we identified, discussed and filled several gaps in existing literature.

**Develop a methodology applicable in real-world scenarios** Much of the current research evaluates on datasets that include very specific activities, such as preparing a sandwich, ironing clothes, and watering the plants that are less relevant for ADL detection. Additionally, the majority of these datasets are collected in a controlled environment and as such the execution of the activities is very similar among different participants. Moreover, the data collection is often either chunked, meaning each activity is performed separately, or the sequence of activities is predefined. Often, when object tagging is involved, participants are restricted to using specific objects that are placed in a specific place in the environment. This conceals several challenges, such as performing activities in a natural, diverse manner and order, as well as performing activities simultaneously.

In our work we address exactly these shortcomings: we developed our model on *data that was collected without interruptions, without scripting and without observation*. People performed the activities they wanted to, whenever and in the way they wanted to, using the objects they saw fit for performing the activity. The labels are provided by the participants themselves, which introduces an additional challenge, i.e., people interpret the execution of activities in different ways. Whereas some consider certain activities to be happening in parallel, such as going to the toilet while cooking, others see the activity of toileting as an interruption of the cooking activity. This shows that there will be different preferences when such an ADL detection platform is deployed in the real world.

Another delta of our work compared to the current state of the art is that we define this problem as a *multilabel problem* and hence can detect more than one activity at a time. This is important since ADL are very often performed in parallel. We employ an LSTM architecture using intuitive features, which allows the LSTM to keep track of various states concerning the person (through the low-level activity probabilities) and the house (through the features extracted from the ambient sensors).

We make use of continuous sensor data even if there are no labels available for certain time regions within a sample. The fact that we use densely labeled data and incorporate weights along the time dimension in the loss function, allows us to use sensor data that has not been labeled. As such we can use continuous data, instead of limiting it to only labeled periods and throwing away the rest. It is essential to present continuous data to an LSTM if one wants it to learn temporal aspects of the data. This is something that has not been used or addressed previously in the application of ADL detection, as most of the available datasets are collected in controlled environments and the data is made available in sometimes even pre-segmented labeled chunks. Additionally, this approach is suitable for potential personalization through incremental and active learning. In such a scenario we cannot expect the person to always provide labels for a full 10 min at a time. An approach that relies on only labeled data, would throw away not only unlabeled data, but also labeled periods that are shorter than the chosen sample window.

**Research the potential of smartphones for ADL detection** Smartphone is a device that is nowadays affordable and omnipresent. Current research uses and relies a lot on other ambient sensors, such as location sensors, RFID tags, and even motion sensors on objects for their methodologies. However, installation, maintenance and usage of these can be costly and as already discussed in previous work, impractical. We therefore build different modules for extracting useful features from various phone sensors, such as accelerometer, screen status, application usage, step detection and ambient light. We highlight the potential of using only smartphone for detecting ADL, such as preparing meal, toileting, and watching TV among others. Our results show that this is possible. With using only the probabilities of the low-level activities as a feature, we obtain a TPR and a balanced accuracy of above 50% for all of the 12 activities. Our results show that accelerometer is the most valuable phone sensor for detecting the ADL of interest. Additional phone modalities such as phone usage, step count and ambient light, bring only marginal improvement of TPR of detecting toileting, eating and walking. This is not very surprising as these sensors do not hold specific information valuable for detecting the majority of activities. One would expect though that the step detector should positively influence the detection of walking (which is indeed the case) and possibly the room transition. It seems however, that the predictions for walking from low-level HAR detection model are enough and make the step detector obsolete. Additionally, the phone usage feature should bring improvement in the detecting of the using phone activity, but this is not the case. This might be a result of certain limitations of our study which are discussed in section “Additional challenges and limitations of our work”, and not the feature being uninformative.

**Research the added value of ambient sensors for ADL detection** Regardless of the limitations mentioned before, ambient sensors can be valuable for improving the detection accuracy. Some sensors are adequate for real-world applications and we should not ignore their potential. We therefore researched the potential of incorporating different combinations of ambient sensors. We constructed meaningful features from these sensors for ADL detection and incorporated them with the features extracted from the phone. Our results, presented in section “Probabilities and location”, show that room-level location sensor leverages the largest accuracy. Specifically, this sensor improves the results for activities such as preparing drink and meal, toileting and eating. For the first two we observe an improvement of 27% and 16% in the TPR respectively, 17% for toileting and 13% for eating. For all of these activities there is an improvement in balanced accuracy as well. These activities might be, in real-world applications, such as elderly monitoring, of greater importance than activities such as using computer, or using phone. Adding more ambient sensors brings only marginal to no improvement to the accuracy of the models as can be seen in Supplementary Table 3 and Supplementary Table 6. These results indicate that it may be better to invest in good and accurate room-level location sensor, before thinking of incorporating other potentially more expensive and more difficult to maintain ambient sensors.

### Additional challenges and limitations of our work

There are several limitations to our work that we would like to discuss. First, we want to reflect on the fact that all the data is collected in the same house. Even though we have data from many participants, and they performed their activities in full freedom, it is still true that all of this data collection was performed in the same house that was not their personal home. Aside for the possibility that people might be more swift and comfortable performing activities in their own home, the setup of their houses would be also different. It is now the case that there is only one TV present and that is in the living room of this house. In other houses there might be several TVs in different locations, such as living room and bedroom. Similarly there are three toilets in the house, and all appliances are at the same place for all participants. The layout and distance between different room locations is of course the same for every participant, which would not be the case in different houses. Even though we have chosen the features from all sensors to be as generic, and therefore transferable, as possible, there might be certain aspects of these that will be correlated to the layout of the house so future research should evaluate robustness of our methodology across different houses.

A second challenge and limitation is the labelling process. Even though self-reporting has advantages for developing more robust models and gaining insight in people’s differences and preferences, it poses certain limitations. For example shorter activities, such as room transition are totally ignored by some participants, and for others they are shifted in time due to labelling imprecision. Sometimes the actual room transition activity happens well before, or well after the labelled period. It is understandable that as some room transitions last only several seconds, participants will indicate an approximate rather than the exact period of time. As previously mentioned in section “Label handling” we had to make certain choices to pre-process the labels. Some activities that were labelled together under the same period of time were kept together as activities performed in parallel, such as eating and watching TV or working on computer and using the phone. Others, such as room transition and toileting, were split and the start and end time were adjusted, giving 30 s of time for the room transition. Lastly, some were removed from the label, such as preparing meal in a label that included both preparing meal and toileting. We consider that a person can in such a case engage actively in only one of those activities, and that would be the toileting in the given example.

A third limitation is the fact that we did not have user-given low-level labels. We obtained the labels by predicting the activities with a pre-trained model using wrist-worn wearable data. So our phone low-level HAR detection model is limited by the accuracy and the biases by the wearable model. However, the least accurately predicted activity, lying, is possibly included in only few ADLs, such as watching TV, using phone and other. In general the results we obtain are acceptable, allowing us to further build on the predictions obtained with this model.

A fourth limitation is that processing the features for phone usage based on applications and screen status is challenging. The data is collected using different smartphones, with different android versions and settings. As such, for some people the phone might never go in locked state, for others the screen would go in ON state and additionally an application would be pushed to foreground even if they only receive a notification. Additionally, several people refused the data collection of their phone usage. For these people we do not have application usage and screen status data. This could have a negative impact on the model that includes the phone usage in the combination of features, as for these participants this feature is calculated to be false, also in the samples labeled as using phone.

As a fifth limitation, in our work we consider only 5 data modalities from the smartphone: accelerometer, step detector, screen status, application usage and ambient light. However, smartphones are equipped with more sensors: gyroscope, magnetometer, linear accelerometer, proximity, keyboard strokes, etc. These sensors may yield additional information and value for detecting ADL if incorporated. Additionally, we adopt the accelerometer only in an indirect way, namely to calculate the probabilities of the low-level activities. This might lead to loss of information on the movement when performing different ADL, and thus future work could focus on developing a model that uses the raw accelerometer, and/or additional higher frequency motion sensors, which may lead to superior performance.

Last but not least we did not apply any post processing, such as label smoothing. There is one prediction every 6 s, which is quite frequent, so a label smoothing over a longer period, such as 30 s might improve the results.

## Conclusion and future work

In this work we propose a data-driven approach for ADL detection using an LSTM architecture. We research the potential of the smartphone as the main source of data, and results show that, even using only accelerometer data from the phone, we can achieve satisfactory results. We moreover show that incorporating ambient sensors yields to improvement of the results, but not all sensors are strictly necessary, and the room-level location sensor is empirically shown to be the most valuable ambient sensor. Our findings can be useful and informative for applications of ADL detection in real-world scenarios, such as elderly care.

We use a single model that can detect different activities, regardless of the location (but location info improves accuracy of the model), duration or the objects employed during execution. Our approach can additionally detect activities that happen concurrently, which happens often in real-world scenarios, but has not been researched enough. As we use the smartphone as a main data source, our methodology can be easily employed in different house setups with different ambient sensors available.

As future work we identify the possibility to use extra smartphone sensors, such as gyroscope and magnetometer as these could hold more detailed information for movements specific to certain activities. We additionally think that it is worth researching the potential of using raw motion sensor data directly for ADL detection, instead of the hierarchical approach we have adopted in this work, i.e. first calculating probabilities of low-level activities and using those as features for the ADLs detection. There are also other ambient sensors, such as contact sensors in cupboards, humidity sensor in the bathroom, and tap water utilisation among others, that are left as future research to evaluate there potential on ADL detection performance. Last but not least, the list of activities can be extended and include more activities such as sleeping, cleaning and exercising.

So, it is clear, detecting activities of daily living remains an interesting challenge to optimize care in an ageing population, with numerous obstacles yet to be overcome. However, we’ve tried with this manuscript to achieve some important strides in this research area.

### Supplementary Information

Supplementary Information.

## Data Availability

The data used in this work can be found at: https://dahcc.idlab.ugent.be/.
