# Supplementary material for: Unlocking the potential of smartphone and ambient sensors for ADL detection
Source: Sci Rep. 2024 Mar 5;14:5392. doi: 10.1038/s41598-024-56123-0 (PMC10914737; doi:10.1038/s41598-024-56123-0)
Supplement: Supplementary file 1 — Supplementary Information. [file 41598_2024_56123_MOESM1_ESM.pdf]

1 Supplementary information

1.1 Low-level activity probabilities, phone use, and steps

	LogLoss	Balanced Accuracy	F1-macro	F1-micro	TPR	TNR
preparing drink	0.42 ± 0.07	0.69 ± 0.15	0.51 ± 0.03	0.86 ± 0.02	0.51 ± 0.30	0.86 ± 0.02
preparing meal	0.36 ± 0.10	0.75 ± 0.12	0.65 ± 0.08	0.87 ± 0.04	0.60 ± 0.28	0.90 ± 0.04
toileting	0.50 ± 0.19	0.82 ± 0.13	0.56 ± 0.09	0.79 ± 0.09	0.84 ± 0.20	0.79 ± 0.09
eating	0.76 ± 0.07	0.51 ± 0.08	0.46 ± 0.08	0.61 ± 0.11	0.36 ± 0.15	0.66 ± 0.13
using computer	0.64 ± 0.13	0.65 ± 0.14	0.64 ± 0.11	0.68 ± 0.08	0.61 ± 0.25	0.70 ± 0.09
watching tv	0.90 ± 0.20	0.62 ± 0.10	0.50 ± 0.07	0.64 ± 0.10	0.58 ± 0.25	0.66 ± 0.13
walking	0.22 ± 0.15	0.94 ± 0.04	0.78 ± 0.10	0.94 ± 0.03	0.95 ± 0.08	0.94 ± 0.03
showering	0.16 ± 0.09	0.93 ± 0.04	0.67 ± 0.12	0.93 ± 0.04	0.84 ± 0.29	0.93 ± 0.04
organizing	0.69 ± 0.15	0.73 ± 0.16	0.47 ± 0.06	0.69 ± 0.13	0.78 ± 0.29	0.68 ± 0.15
using phone	0.78 ± 0.28	0.65 ± 0.13	0.49 ± 0.15	0.64 ± 0.20	0.66 ± 0.33	0.65 ± 0.24
room transition	0.70 ± 0.07	0.61 ± 0.13	0.45 ± 0.07	0.65 ± 0.07	0.52 ± 0.29	0.69 ± 0.07
other	0.74 ± 0.16	0.65 ± 0.09	0.55 ± 0.10	0.63 ± 0.13	0.68 ± 0.15	0.62 ± 0.16
mean ± std	0.57 ± 0.24	0.71 ± 0.13	0.56 ± 0.10	0.74 ± 0.13	0.66 ± 0.17	0.76 ± 0.12

Supplementary Table 1. Results of the ADL detection model, trained on probabilities of low-level activities, phone usage, and step count. Mean and std across the 10 participants in the holdout set for the different performance metrics are shown.

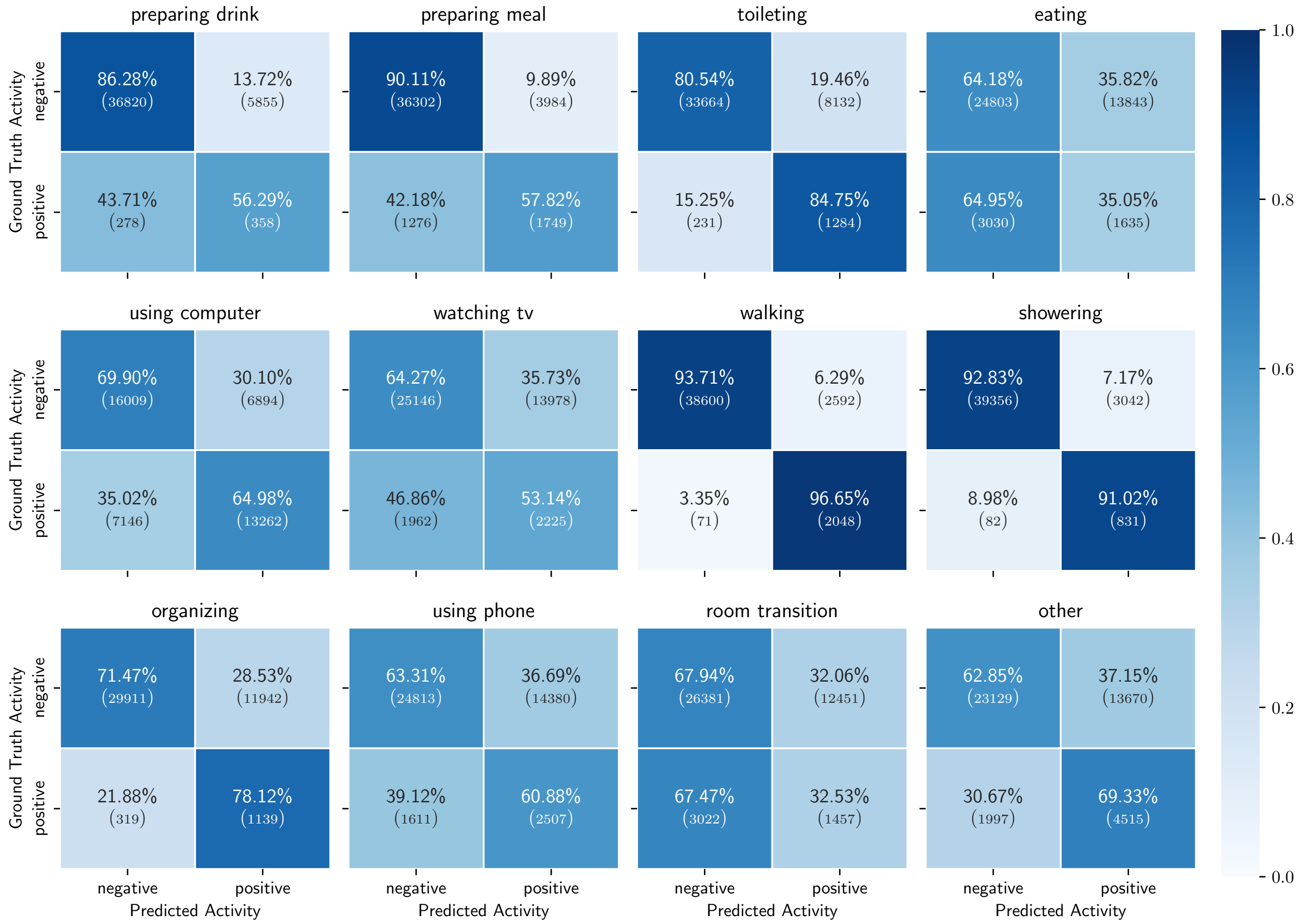

Supplementary Figure 1. Binary confusion matrices of the results of the ADL detection model, trained on probabilities of low-level activities, phone usage, and step count.

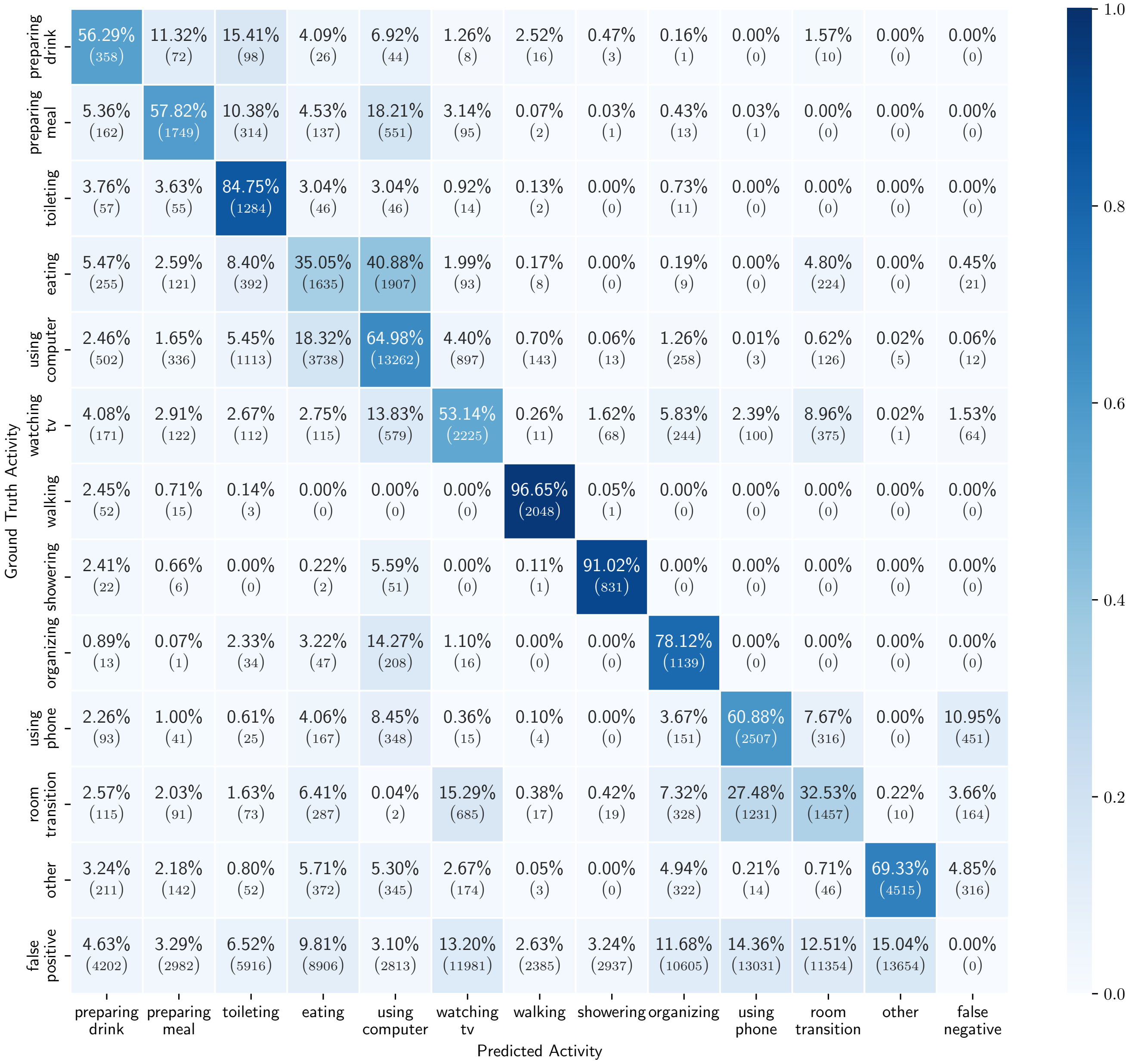

Supplementary Figure 2. Confusion matrix of the results of the ADL detection model, trained on probabilities of low-level activities, phone usage, and step count.

1.2 Low-level activity probabilities, phone use, steps, and ambient light from phone

In this section we show the results obtained when using all features we extracted from the phone sensors. Supplementary Table 2 shows the mean and std of the performance metrics for each activity across the 10 participants in the holdout set. The binary and full confusion matrix can be found in Section 1.2. We do not observe any particular improvement in the results compared to using only low-level activities probabilities, except for detecting watching TV and distinguishing better eating from using computer. However, the accuracy of detecting activities such as preparing drink, preparing meal and room transition drops considerably. These results indicate that these additional sensors do not have particular added value to detecting the activities we are interested in.

	LogLoss	Balanced Accuracy	F1-macro	F1-micro	TPR	TNR
preparing drink	0.47 ± 0.12	0.61 ± 0.16	0.48 ± 0.03	0.83 ± 0.05	0.38 ± 0.34	0.84 ± 0.05
preparing meal	0.37 ± 0.16	0.75 ± 0.13	0.67 ± 0.09	0.88 ± 0.05	0.59 ± 0.28	0.91 ± 0.04
toileting	0.67 ± 0.44	0.76 ± 0.13	0.51 ± 0.13	0.72 ± 0.18	0.79 ± 0.22	0.72 ± 0.18
eating	0.82 ± 0.10	0.56 ± 0.11	0.44 ± 0.08	0.54 ± 0.11	0.61 ± 0.28	0.52 ± 0.14
using computer	0.60 ± 0.09	0.67 ± 0.14	0.66 ± 0.12	0.70 ± 0.08	0.62 ± 0.28	0.73 ± 0.08
watching tv	0.98 ± 0.16	0.64 ± 0.08	0.43 ± 0.09	0.50 ± 0.10	0.81 ± 0.13	0.46 ± 0.10
walking	0.17 ± 0.13	0.91 ± 0.14	0.82 ± 0.16	0.96 ± 0.03	0.86 ± 0.29	0.97 ± 0.03
showering	0.37 ± 0.35	0.90 ± 0.12	0.60 ± 0.14	0.86 ± 0.17	0.86 ± 0.29	0.85 ± 0.17
organizing	0.62 ± 0.18	0.69 ± 0.13	0.48 ± 0.07	0.72 ± 0.14	0.66 ± 0.31	0.72 ± 0.15
using phone	0.82 ± 0.57	0.67 ± 0.11	0.53 ± 0.15	0.69 ± 0.19	0.65 ± 0.28	0.70 ± 0.22
room transition	0.65 ± 0.16	0.57 ± 0.16	0.45 ± 0.07	0.68 ± 0.15	0.42 ± 0.29	0.73 ± 0.14
other	0.84 ± 0.33	0.62 ± 0.10	0.52 ± 0.14	0.61 ± 0.18	0.61 ± 0.21	0.62 ± 0.23
mean ± *std*	0.62 ± 0.24	0.70 ± 0.12	0.55 ± 0.12	0.72 ± 0.14	0.66 ± 0.16	0.73 ± 0.15

Supplementary Table 2. Results of the ADL detection model, trained on probabilities of low-level activities, phone usage, step count, and ambient light from phone. Mean and std across the 10 participants in the holdout set for the different performance metrics are shown.

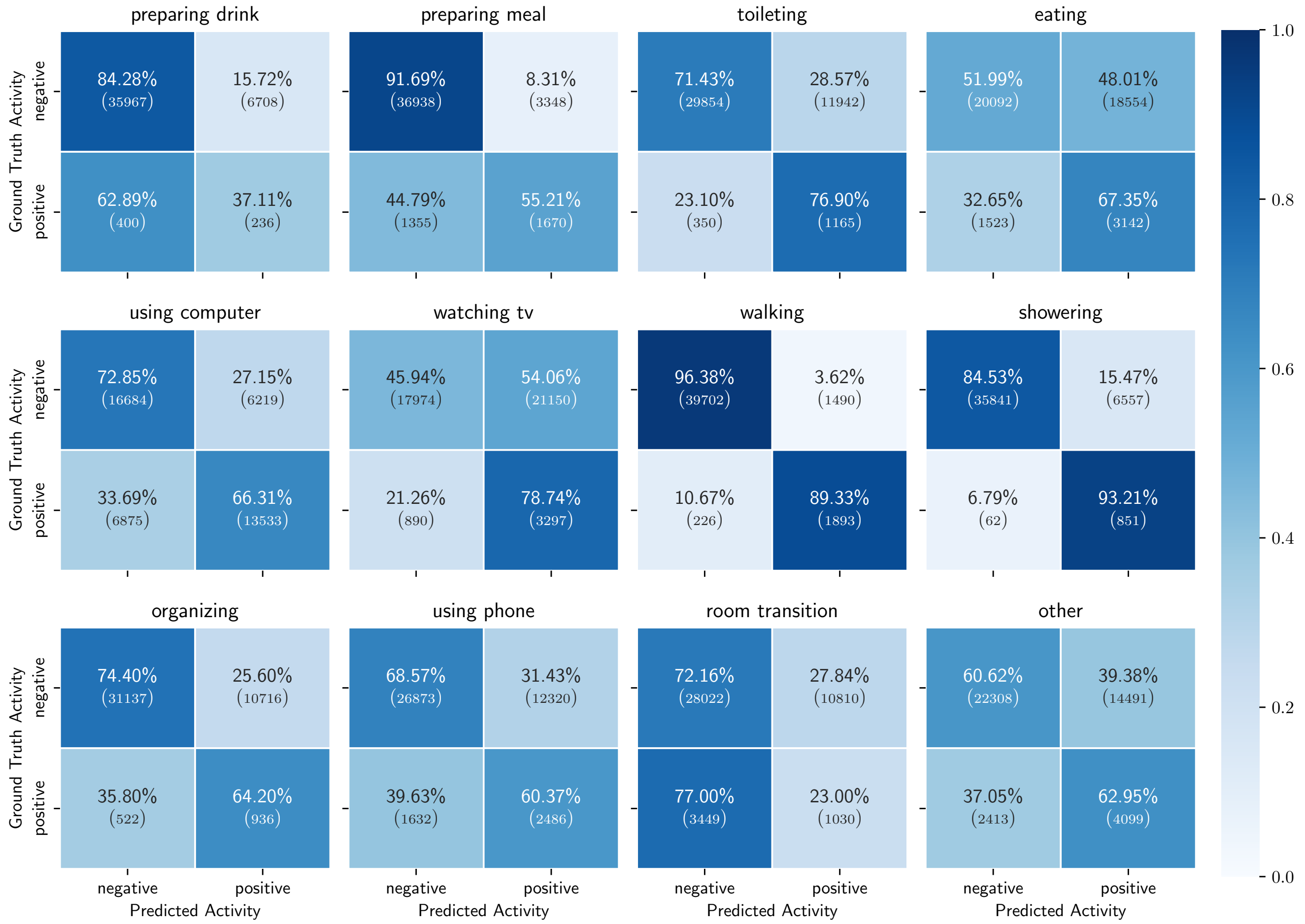

Supplementary Figure 3. Binary confusion matrices of the results of the ADL detection model, trained on probabilities of low-level activities, phone usage, step count, and ambient light from phone.

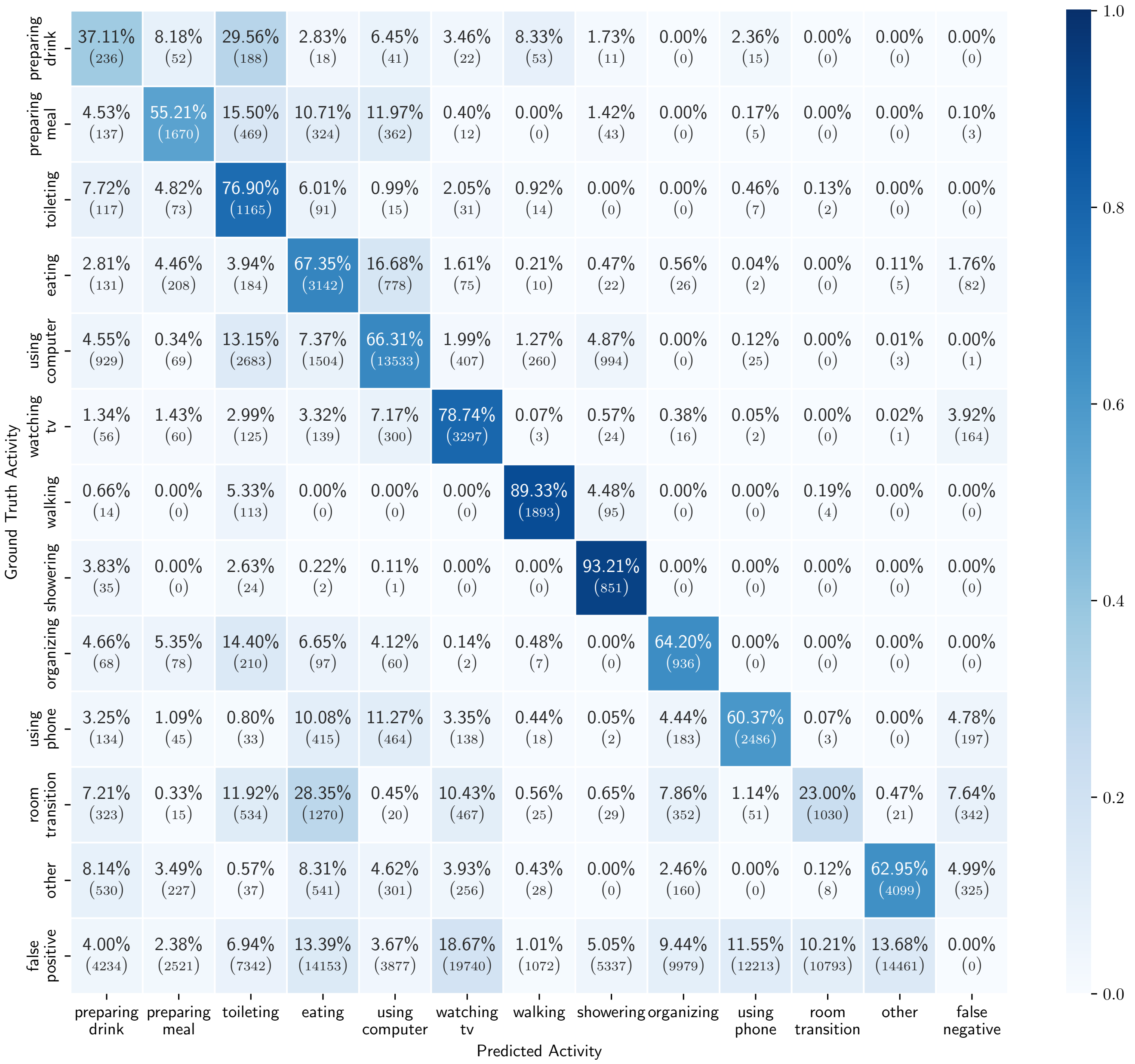

Supplementary Figure 4. Confusion matrix of the results of the ADL detection model, trained on probabilities of low-level activities, phone usage, step count, and ambient light from phone.

Supplementary Fig. 3 shows the confusion matrices for all activities over all the 10 participants in the holdout set, and Supplementary Fig. 4 shows the full confusion matrix.

	LogLoss	Balanced Accuracy	F1-macro	F1-micro	TPR	TNR
preparing drink	0.55 ± 0.30	0.68 ± 0.12	0.46 ± 0.06	0.76 ± 0.17	0.60 ± 0.34	0.76 ± 0.17
preparing meal	0.45 ± 0.19	0.71 ± 0.15	0.60 ± 0.12	0.80 ± 0.11	0.59 ± 0.29	0.82 ± 0.12
toileting	0.71 ± 0.26	0.73 ± 0.12	0.48 ± 0.09	0.70 ± 0.12	0.75 ± 0.24	0.70 ± 0.12
eating	0.75 ± 0.36	0.59 ± 0.10	0.51 ± 0.11	0.68 ± 0.18	0.50 ± 0.34	0.69 ± 0.24
using computer	0.59 ± 0.08	0.74 ± 0.10	0.69 ± 0.13	0.74 ± 0.09	0.73 ± 0.25	0.74 ± 0.12
watching tv	0.71 ± 0.56	0.71 ± 0.13	0.60 ± 0.20	0.72 ± 0.25	0.71 ± 0.28	0.71 ± 0.29
walking	0.30 ± 0.11	0.87 ± 0.13	0.66 ± 0.10	0.88 ± 0.05	0.86 ± 0.31	0.88 ± 0.06
showering	0.30 ± 0.28	0.79 ± 0.26	0.59 ± 0.13	0.87 ± 0.16	0.61 ± 0.45	0.88 ± 0.16
organizing	0.49 ± 0.24	0.62 ± 0.14	0.48 ± 0.09	0.78 ± 0.17	0.46 ± 0.39	0.79 ± 0.19
using phone	0.60 ± 0.25	0.65 ± 0.12	0.56 ± 0.15	0.75 ± 0.19	0.51 ± 0.31	0.78 ± 0.22
room transition	0.91 ± 0.27	0.59 ± 0.11	0.37 ± 0.09	0.51 ± 0.18	0.67 ± 0.38	0.51 ± 0.24
other	0.70 ± 0.18	0.66 ± 0.11	0.57 ± 0.11	0.68 ± 0.13	0.64 ± 0.23	0.69 ± 0.18
mean ± *std*	0.59 ± 0.18	0.10 ± 0.08	0.55 ± 0.09	0.74 ± 0.10	0.64 ± 0.12	0.75 ± 0.10

Supplementary Table 3. Results of the ADL detection model, trained on probabilities of low-level activities, and appliances usage. Mean and std across the 10 participants in the holdout set for the different performance metrics are shown.

1.3 Low-level activity probabilities and appliances usage

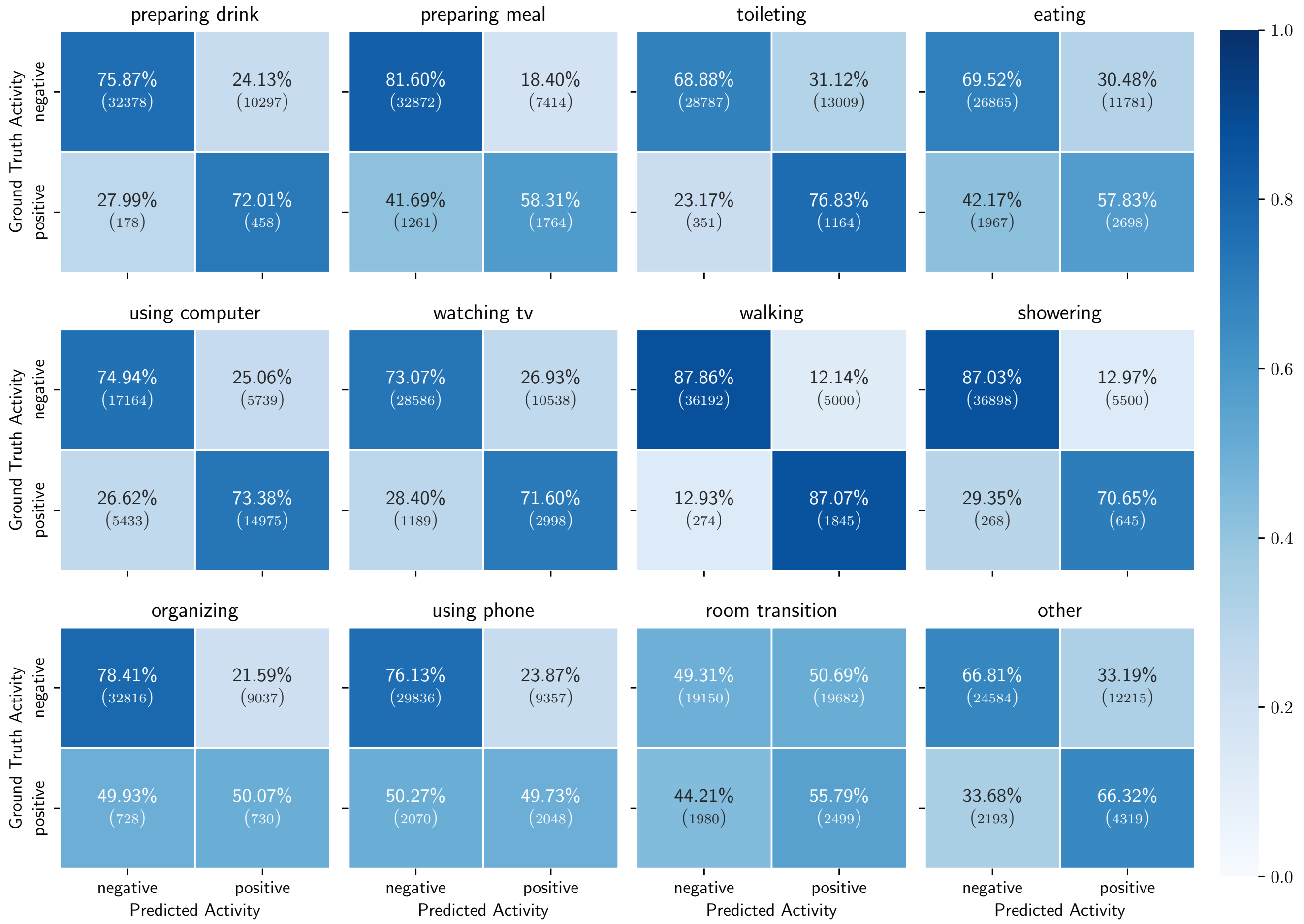

Supplementary Figure 5. Binary confusion matrices of the results of the ADL detection model, trained on probabilities of low-level activities, and appliances usage.

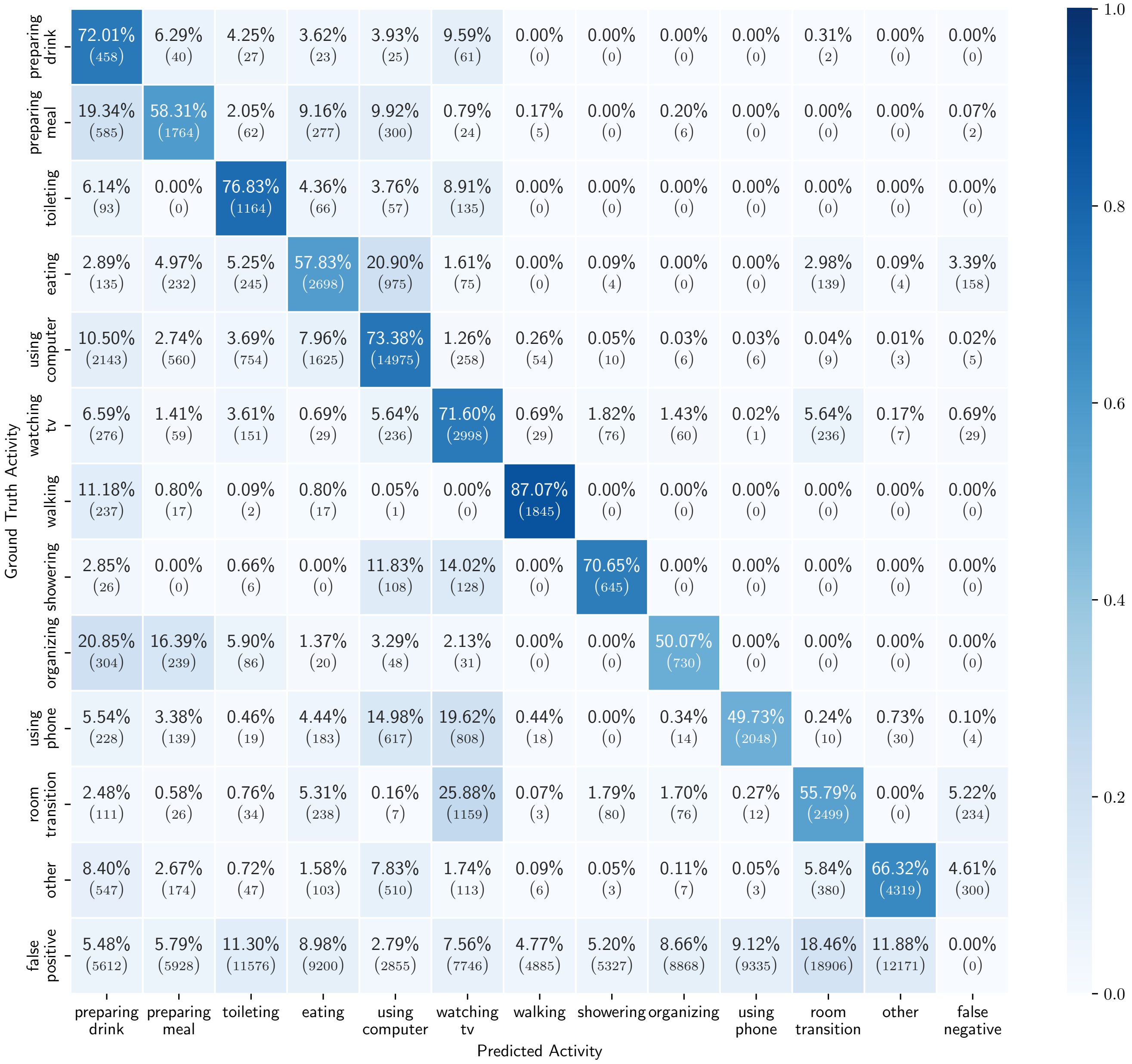

Supplementary Figure 6. Confusion matrix of the results of the ADL detection model, trained on probabilities of low-level activities, and appliances usage.

	LogLoss	Balanced Accuracy	F1-macro	F1-micro	TPR	TNR
preparing drink	0.44 ± 0.14	0.72 ± 0.11	0.54 ± 0.04	0.89 ± 0.07	0.55 ± 0.23	0.89 ± 0.07
preparing meal	0.39 ± 0.16	0.68 ± 0.11	0.63 ± 0.09	0.86 ± 0.09	0.48 ± 0.21	0.89 ± 0.08
toileting	0.25 ± 0.07	0.81 ± 0.11	0.63 ± 0.09	0.89 ± 0.04	0.72 ± 0.21	0.90 ± 0.04
eating	0.67 ± 0.21	0.59 ± 0.10	0.51 ± 0.11	0.70 ± 0.20	0.49 ± 0.39	0.70 ± 0.26
using computer	0.64 ± 0.13	0.68 ± 0.13	0.63 ± 0.14	0.70 ± 0.12	0.81 ± 0.24	0.54 ± 0.24
watching tv	0.55 ± 0.30	0.73 ± 0.11	0.64 ± 0.16	0.76 ± 0.19	0.69 ± 0.25	0.77 ± 0.21
walking	0.21 ± 0.16	0.92 ± 0.07	0.81 ± 0.10	0.96 ± 0.03	0.88 ± 0.15	0.96 ± 0.03
showering	0.10 ± 0.06	0.97 ± 0.02	0.76 ± 0.14	0.97 ± 0.02	0.88 ± 0.29	0.97 ± 0.02
organizing	0.53 ± 0.23	0.58 ± 0.14	0.49 ± 0.06	0.81 ± 0.18	0.35 ± 0.32	0.82 ± 0.19
using phone	0.53 ± 0.12	0.60 ± 0.14	0.54 ± 0.08	0.81 ± 0.08	0.33 ± 0.32	0.87 ± 0.08
room transition	0.68 ± 0.19	0.62 ± 0.13	0.50 ± 0.08	0.74 ± 0.13	0.47 ± 0.36	0.76 ± 0.21
other	0.60 ± 0.11	0.60 ± 0.08	0.58 ± 0.07	0.76 ± 0.08	0.35 ± 0.21	0.84 ± 0.10
mean $\pm$ *std*	0.47 ± 0.19	0.71 ± 0.13	0.61 ± 0.10	0.82 ± 0.09	0.58 ± 0.21	0.83 ± 0.12

Supplementary Table 4. Results of the ADL detection model, trained on all features except location. Mean and std across the 10 participants in the holdout set for the different performance metrics are shown.

1.4 All features except location

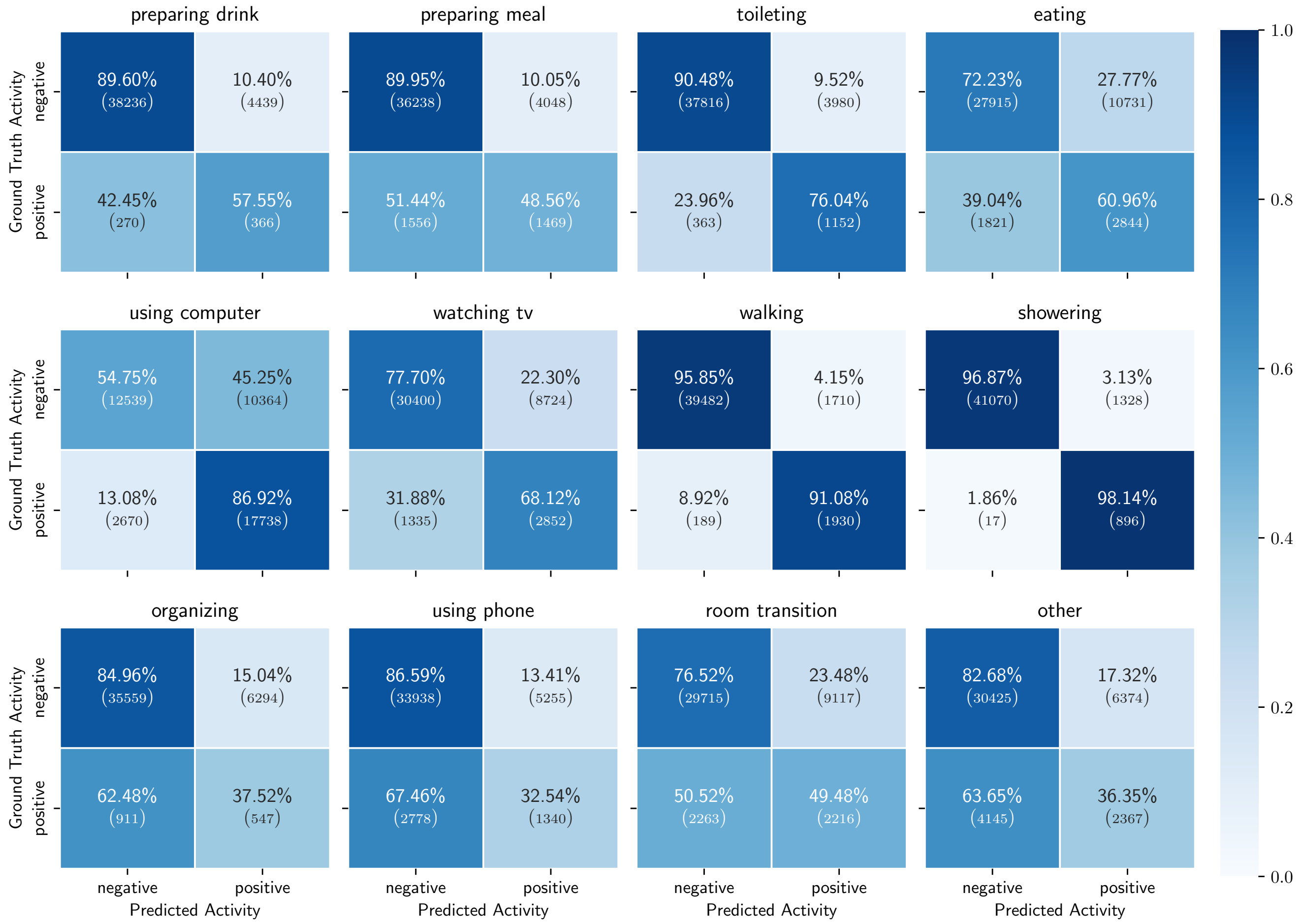

Supplementary Figure 7. Binary confusion matrices of the results of the ADL detection model, trained on all features except location.

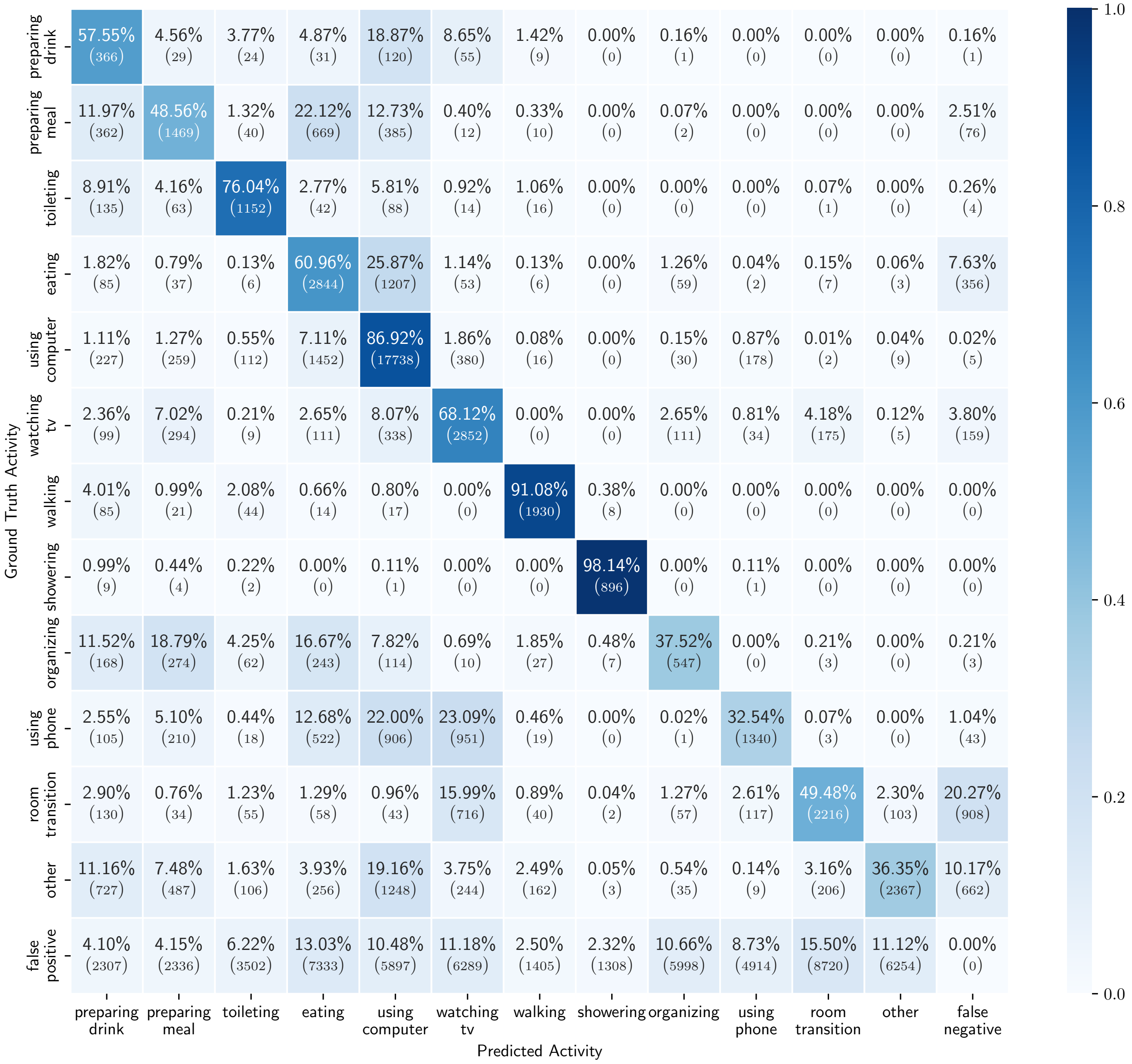

Supplementary Figure 8. Confusion matrix of the results of the ADL detection model, trained on all features except location.

	LogLoss	Balanced Accuracy	F1-macro	F1-micro	TPR	TNR
preparing drink	0.49 ± 0.38	0.80 ± 0.12	0.53 ± 0.09	0.84 ± 0.16	0.76 ± 0.21	0.84 ± 0.16
preparing meal	0.42 ± 0.19	0.85 ± 0.12	0.69 ± 0.09	0.86 ± 0.06	0.84 ± 0.24	0.86 ± 0.06
toileting	0.32 ± 0.08	0.91 ± 0.03	0.66 ± 0.10	0.89 ± 0.05	0.92 ± 0.04	0.89 ± 0.05
eating	0.59 ± 0.26	0.59 ± 0.13	0.51 ± 0.09	0.70 ± 0.17	0.47 ± 0.42	0.71 ± 0.23
using computer	0.53 ± 0.13	0.77 ± 0.08	0.71 ± 0.13	0.76 ± 0.10	0.79 ± 0.22	0.75 ± 0.14
watching tv	0.38 ± 0.22	0.78 ± 0.11	0.72 ± 0.14	0.85 ± 0.13	0.69 ± 0.28	0.87 ± 0.16
walking	0.18 ± 0.11	0.94 ± 0.06	0.79 ± 0.13	0.94 ± 0.04	0.94 ± 0.11	0.94 ± 0.04
showering	0.16 ± 0.11	0.92 ± 0.12	0.75 ± 0.16	0.96 ± 0.04	0.80 ± 0.34	0.96 ± 0.03
organizing	0.67 ± 0.31	0.53 ± 0.08	0.44 ± 0.11	0.72 ± 0.24	0.34 ± 0.35	0.73 ± 0.26
using phone	0.65 ± 0.16	0.63 ± 0.12	0.56 ± 0.12	0.77 ± 0.17	0.44 ± 0.29	0.82 ± 0.20
room transition	0.88 ± 0.32	0.56 ± 0.10	0.38 ± 0.17	0.49 ± 0.25	0.63 ± 0.31	0.49 ± 0.29
other	0.70 ± 0.12	0.67 ± 0.05	0.61 ± 0.07	0.73 ± 0.11	0.58 ± 0.21	0.76 ± 0.15
mean $\pm$ *std*	0.50 ± 0.22	0.75 ± 0.15	0.61 ± 0.13	0.79 ± 0.13	0.68 ± 0.19	0.80 ± 0.13

Supplementary Table 5. Results of the ADL detection model, trained on only features derived from ambient sensors. Mean and std across the 10 participants in the holdout set for the different performance metrics are shown.

1.4.1 Only ambient sensors

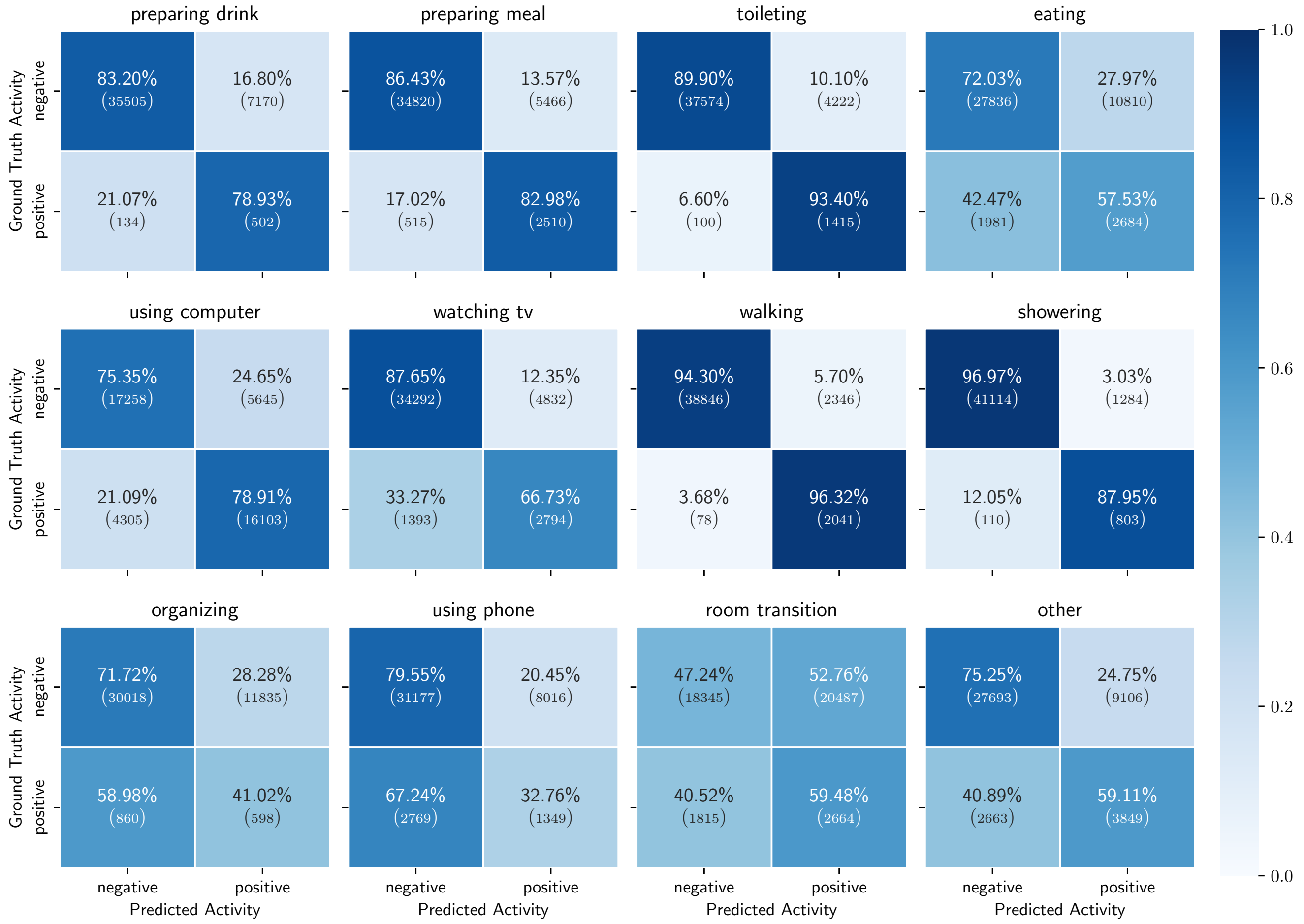

Supplementary Figure 9. Binary confusion matrices of the results of the ADL detection model, trained on only features derived from ambient sensors.)

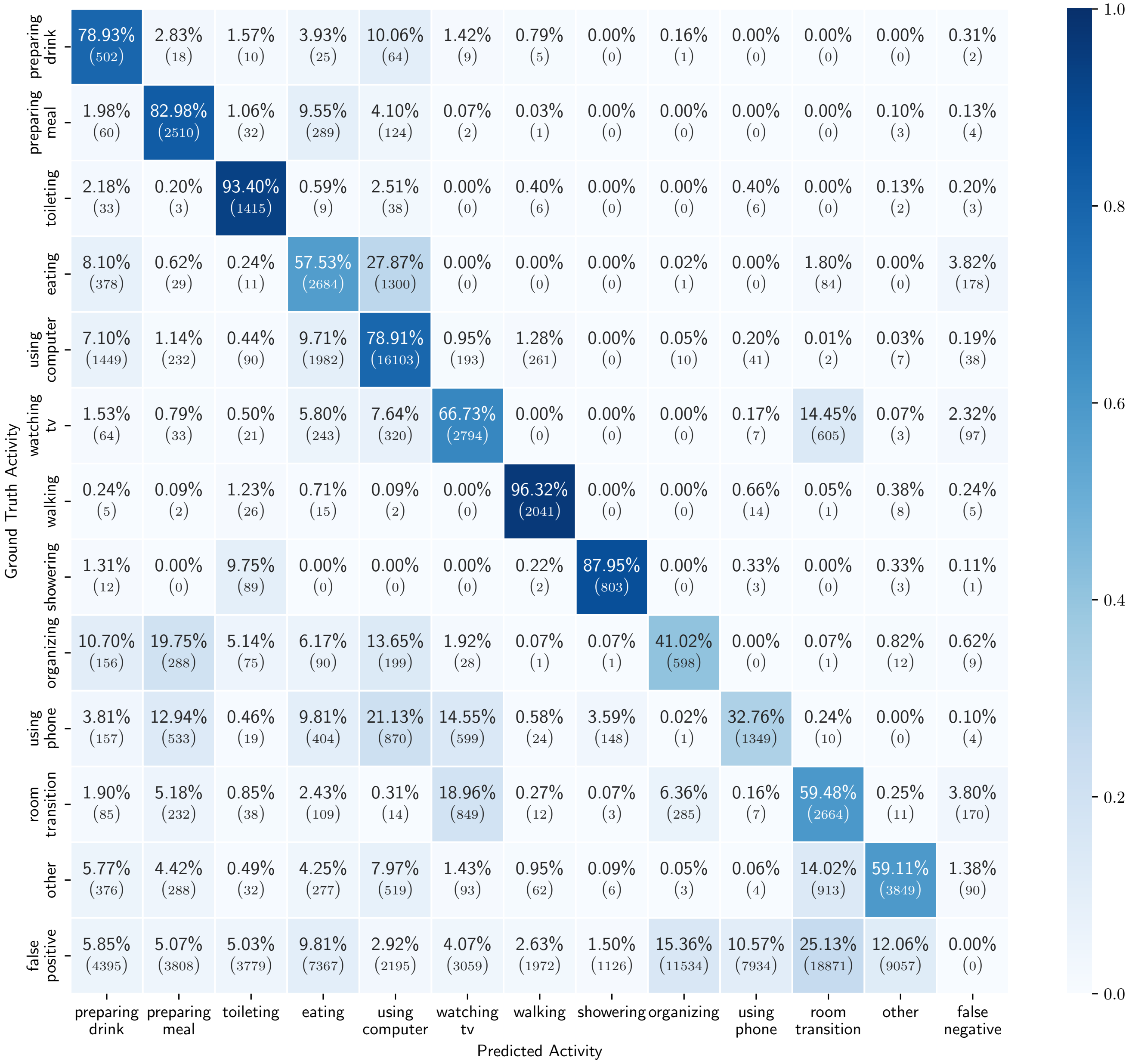

Supplementary Figure 10. Confusion matrix of the results of the ADL detection model, trained on only features derived from ambient sensors.

1.5 All features

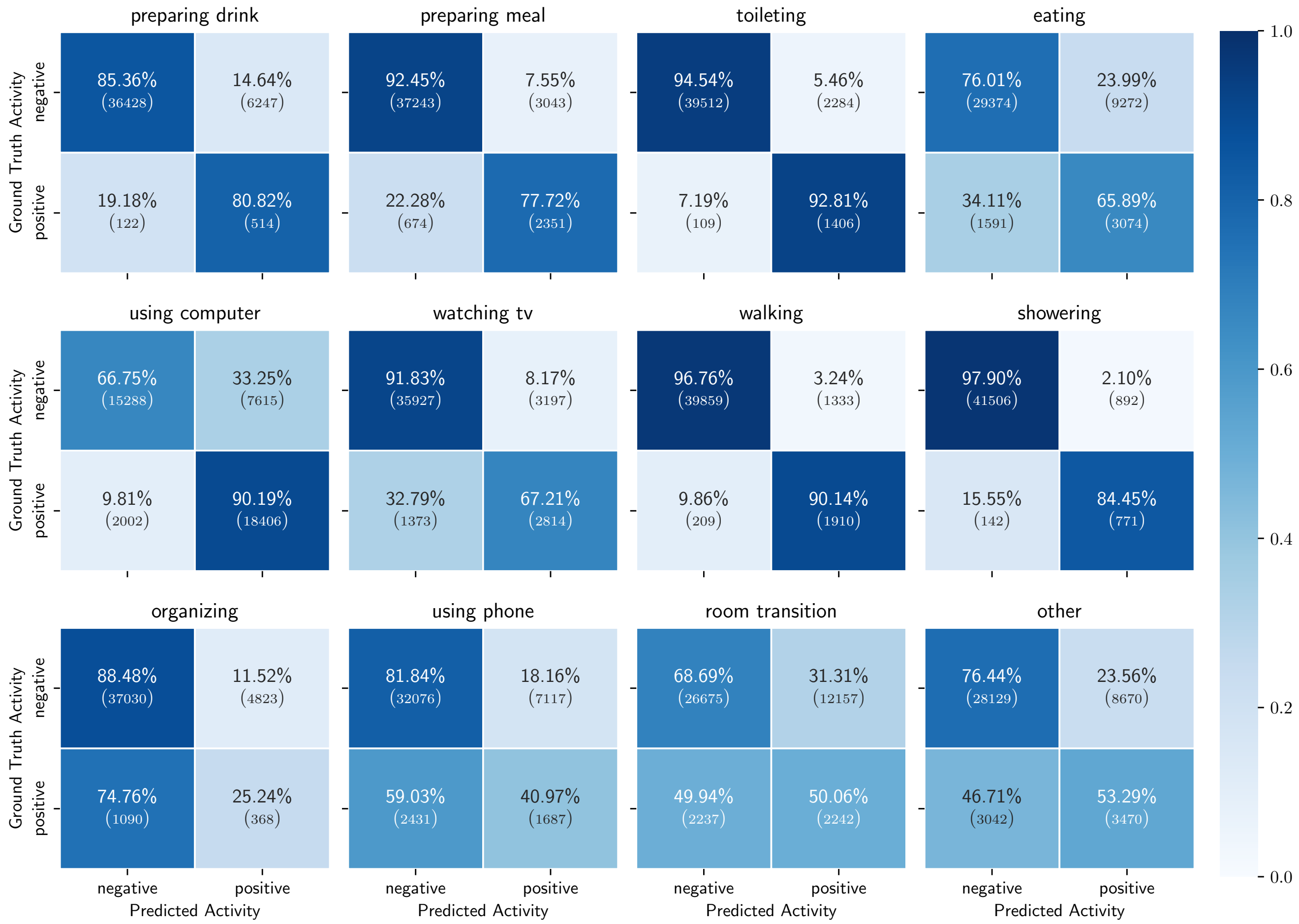

Supplementary Figure 11. Binary confusion matrices of the results of the ADLs model, trained on all features (phone and ambient)

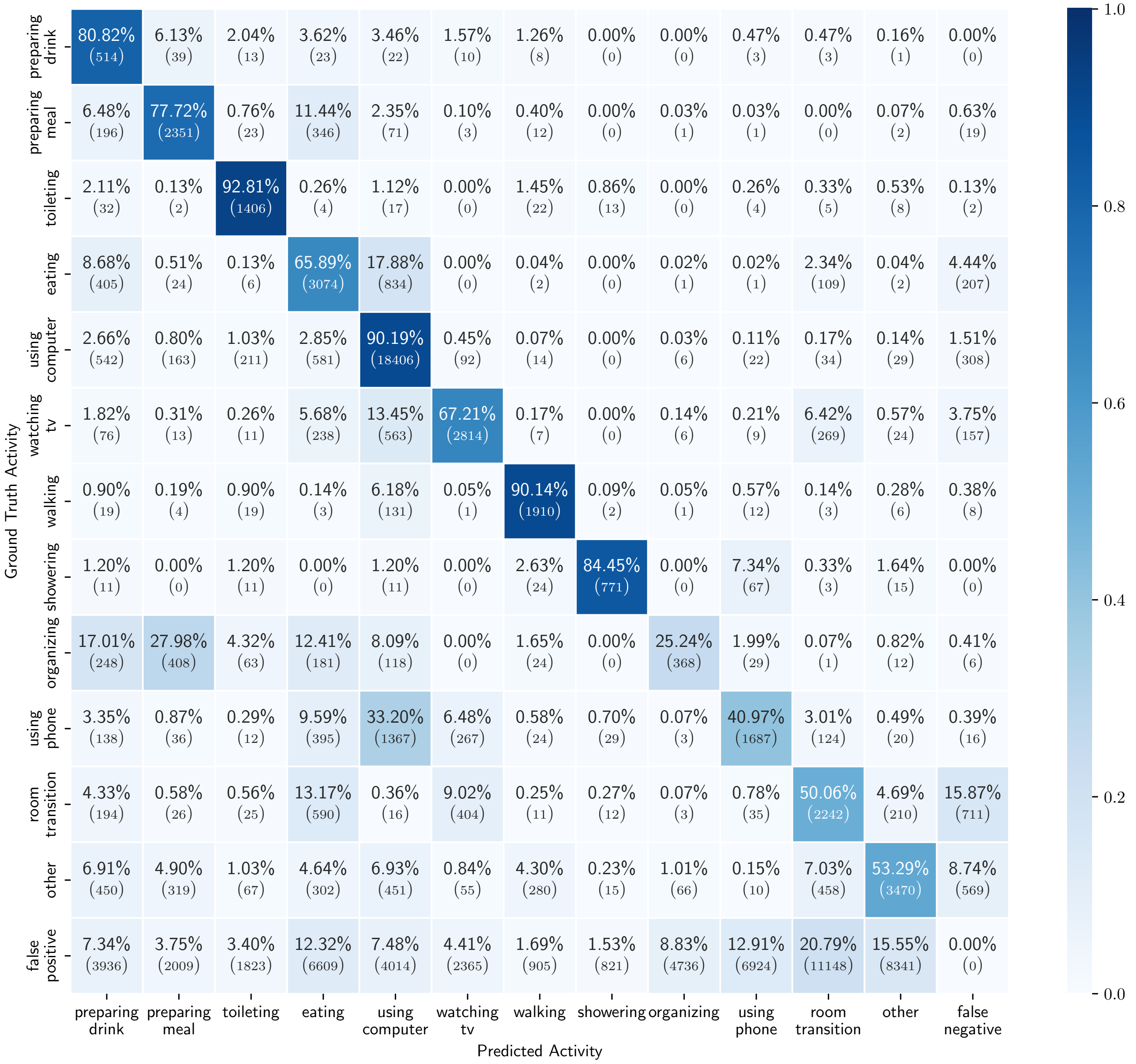

Supplementary Figure 12. Confusion matrix of the results of the ADL detection model, trained on all features (phone and ambient).

In this section, we present the results obtained using all sensors. Supplementary Table 6 contains the mean and std of the performance metrics for each activity, and Supplementary Fig. 11 shows the confusion matrices. Compared to using only ambient features, we observe marginal differences. Whereas we see significant improvement in detecting eating, the model seems to still be struggling with organizing, using phone and room transition. In Supplementary Fig. 12 we can see that organizing is often mistaken for the same activities as when using only ambient sensors. Similarly for using phone, whereas it improves from only using ambient sensors, it does not reach the accuracy from when we use only the low-level probabilities as features. This indicates that maybe, there are some features from the ambient sensors that have a negative impact on the using phone detection.

	LogLoss	Balanced Accuracy	F1-macro	F1-micro	TPR	TNR
preparing drink	0.45 ± 0.29	0.79 ± 0.14	0.54 ± 0.07	0.86 ± 0.13	0.73 ± 0.26	0.86 ± 0.13
preparing meal	0.25 ± 0.09	0.85 ± 0.10	0.75 ± 0.07	0.91 ± 0.04	0.78 ± 0.19	0.92 ± 0.04
toileting	0.24 ± 0.11	0.93 ± 0.03	0.74 ± 0.09	0.94 ± 0.03	0.91 ± 0.05	0.94 ± 0.03
eating	0.57 ± 0.31	0.65 ± 0.15	0.59 ± 0.15	0.75 ± 0.18	0.54 ± 0.36	0.76 ± 0.24
using computer	0.55 ± 0.16	0.77 ± 0.06	0.73 ± 0.10	0.78 ± 0.08	0.88 ± 0.11	0.67 ± 0.11
watching tv	0.31 ± 0.15	0.81 ± 0.12	0.76 ± 0.11	0.89 ± 0.07	0.69 ± 0.27	0.92 ± 0.08
walking	0.16 ± 0.19	0.91 ± 0.14	0.82 ± 0.15	0.96 ± 0.03	0.85 ± 0.28	0.97 ± 0.03
showering	0.10 ± 0.08	0.92 ± 0.15	0.76 ± 0.17	0.97 ± 0.02	0.77 ± 0.39	0.98 ± 0.02
organizing	0.39 ± 0.23	0.57 ± 0.13	0.53 ± 0.08	0.86 ± 0.13	0.26 ± 0.23	0.89 ± 0.13
using phone	0.58 ± 0.30	0.69 ± 0.14	0.58 ± 0.10	0.78 ± 0.13	0.55 ± 0.32	0.82 ± 0.15
room transition	0.86 ± 0.35	0.64 ± 0.15	0.47 ± 0.11	0.67 ± 0.15	0.59 ± 0.35	0.69 ± 0.19
other	0.67 ± 0.18	0.64 ± 0.07	0.59 ± 0.04	0.72 ± 0.08	0.53 ± 0.15	0.75 ± 0.10
mean	0.43 ± 0.23	0.76 ± 0.12	0.66 ± 0.12	0.84 ± 0.10	0.67 ± 0.19	0.85 ± 0.11

Supplementary Table 6. Results of the ADL detection model, trained on all features (phone and ambient). Mean and std across the 10 participants in the holdout set for the different performance metrics are shown.

"com.android.systemui"
"android"
"com.samsung.android.app.aodservice"
"com.samsung.android.app.cocktailbarservice"
"com.miui.aod"
"com.google.android.inputmethod.latin"
"com.sec.android.inputmethod"

Supplementary Table 7. List of system utilities that we filter out when calculating the using phone feature
